# Comparison of Rates of Type 2 Diabetes in Adults and Children Treated With Anticonvulsant Mood Stabilizers

**DOI:** 10.1001/jamanetworkopen.2022.6484

**Published:** 2022-04-06

**Authors:** Jenny W. Sun, Jessica G. Young, Aaron L. Sarvet, L. Charles Bailey, William J. Heerman, David M. Janicke, Pi-I Debby Lin, Sengwee Toh, Jason P. Block

**Affiliations:** 1 Department of Population Medicine, Harvard Medical School and Harvard Pilgrim Health Care Institute, Boston, Massachusetts; 2 Department of Pediatrics, Children’s Hospital of Philadelphia, Philadelphia, Pennsylvania; 3 Department of Pediatrics, Vanderbilt University Medical Center, Nashville, Tennessee; 4 Department of Clinical and Health Psychology, University of Florida, Gainesville

## Abstract

**Importance:**

Anticonvulsant mood stabilizer treatment is associated with an increased risk of weight gain, but little is known about the risk of developing type 2 diabetes (T2D).

**Objective:**

To evaluate the comparative safety of anticonvulsant mood stabilizers on risk of T2D in adults and children by emulating a target trial.

**Design, Setting, and Participants:**

This observational cohort study used data from IBM MarketScan (2010-2019), with a 5-year follow-up period. The nationwide sample of US commercially insured patients included children (aged 10-19 years) and adults (aged 20-65 years) who initiated anticonvulsant mood stabilizer treatment. Data were analyzed from August 2020 to May 2021.

**Exposures:**

Initiation and continuation of carbamazepine, lamotrigine, oxcarbazepine, or valproate.

**Main Outcomes and Measures:**

Onset of T2D during follow-up. Weighted pooled logistic regression was used to estimate the association of initiation and continuation of carbamazepine, lamotrigine, oxcarbazepine, or valproate with the risk of developing T2D. Inverse probability weights were used to control for confounding and loss to follow-up by measured baseline and time-varying covariates.

**Results:**

The analysis included 274 206 adults (159 428 women [58%]; mean [SD] age, 39.9 [13.2] years) and 74 005 children (38 672 girls [52%]; mean [SD] age, 15.6 [2.6] years) who initiated an anticonvulsant mood stabilizer. In adults, initiation of valproate was associated with an increased risk of developing T2D compared with initiation of lamotrigine (5-year risk difference [RD], 1.17%; 95% CI, 0.66% to 1.76%). The number needed to harm was 87 patients initiating valproate for 1 patient to develop T2D within 5 years compared with initiation of lamotrigine. Point estimates were similar when evaluating the association of treatment continuation (5-year RD, 1.99%; 95% CI, −0.64% to 5.31%). The estimated association was smaller and more variable comparing carbamazepine and oxcarbazepine to lamotrigine. In children, RDs were much smaller and more variable (5-year RD for initiation of oxcarbazepine vs lamotrigine, 0.29%; 95% CI, −0.12% to 0.69%; 5-year RD for initiation of valproate vs lamotrigine, 0.18%; 95% CI, −0.09% to 0.49%).

**Conclusions and Relevance:**

In this cohort study, valproate was associated with the highest risk of developing T2D in adults. The comparative safety was generally similar in children, but estimates were small and variable. In the absence of randomized trials, emulating target trials within health care databases can generate the age-specific drug safety data needed to inform treatment decision-making.

## Key Points

**Question:**

Is treatment with anticonvulsant mood stabilizers associated with an increased risk of type 2 diabetes (T2D)?

**Findings:**

In this cohort study of 274 206 adults and 74 005 children, valproate was associated with the highest risk of developing T2D in adults, with a number needed to harm of 87 patients initiating valproate for 1 patient to develop T2D within 5 years compared with initiation of lamotrigine. In children, findings were generally similar but less precise.

**Meaning:**

The choice of which anticonvulsant mood stabilizer to initiate may be associated with meaningful reductions in T2D incidence, and patients and clinicians concerned about the potential metabolic adverse effects could consider initiating lamotrigine.

## Teaser

This cohort study evaluates the association between anticonvulsant mood stabiliziers and rates of type 2 diabetes in adults and children using data from a nationwide sample of US commercially insured patients.

## Introduction

Over the past several decades, the prevalence of type 2 diabetes (T2D) has increased in the US and globally.^1,2,3,4^ Approximately 1 in 10 people in the US were living with T2D from 2013 to 2016,^1,5^ and youth-onset T2D has increased in the US by approximately 5% annually from 2002 to 2015.^5,6,7^ There are several known risk factors for T2D, including lifestyle, environmental, and psychosocial factors that are key focus areas for prevention.^8^ Patients and clinicians would also benefit from guidance on choosing between similarly effective medications that may have different metabolic adverse effects.

Several long-term medications are associated with increased risk of T2D,^9,10,11^ including some anticonvulsant mood stabilizers. However, little is known about differential risk of T2D associated with specific medications in this class. Anticonvulsant mood stabilizers are widely used by adults and children for the treatment of epilepsy, bipolar disorder, and neuropathic pain.^12,13,14,15,16,17^ In the early 2010s, approximately 1% of adolescents and 5% of adults in the US were treated with an anticonvulsant medication.^18,19^ Weight gain is a known adverse effect for some anticonvulsant mood stabilizers (valproate and carbamazepine),^20,21,22^ but other mood stabilizers are not associated with weight gain (lamotrigine)^21,23,24,25,26^ or have an unclear association with weight (oxcarbazepine). There is some evidence that valproate may be associated with an increased risk of insulin resistance or T2D,^27,28,29,30,31,32^ but most of these studies have been small, and a comprehensive study of comparative risk across commonly used anticonvulsant mood stabilizers has not been conducted. Patients receive anticonvulsant mood stabilizer treatment across the life course, with many initiating during childhood; limited data are available on metabolic risk in children. Age-specific drug safety data are needed to inform treatment decision-making.

A sufficiently large randomized clinical trial (RCT) to evaluate risk of T2D among patients treated with anticonvulsant mood stabilizers would provide the relevant evidence but would be too long and costly to conduct. In the absence of an RCT, evidence can be generated by emulating the experiment (the target trial) within health care databases.^33,34^ The primary goal of this target trial approach is to provide a structured framework for making inferences from observational studies that is transparent about the study question and analytical goal, helping to avoid common methodological pitfalls.^35,36^ Following this approach, we conceptualized the hypothetical trials that we would have liked to conduct; subsequently, we used routinely collected health care utilization data to mimic the trials as closely as possible. The objective of this cohort study was to emulate target trials evaluating the association between anticonvulsant mood stabilizers and incident T2D across the life course.

## Methods

### Specifying the Target Trial Protocol

Outlining the protocol of the hypothetical trials allows clear specification of the study question of interest. We conceptualized 2 target trials: a trial of adults (aged 20-65 years) and a trial of children (aged 10-19 years). Unless otherwise noted, the target trial protocol was the same for adults and children (Table 1). Studying patients across a large age range is important because the association of mood stabilizer treatment with metabolic risk may exist across the life course. This study was approved by the institutional review board of Harvard Pilgrim Health Care as non human subjects research, which waived the need for informed consent, and followed the Strengthening the Reporting of Observational Studies in Epidemiology (STROBE) reporting guideline.

**Table 1.** Overview of Target Trials and Emulation

Protocol component	Target trial specification^a^	Implementation of emulation
Eligibility criteria	Inclusion: Age 20-65 y for adult trial Age 10-19 y for pediatric trial Continuous enrollment in a health plan for at least 1 y Exclusion: No anticonvulsant medication use during the prior year No diagnosis of diabetes (type 1, type 2, secondary or gestational diabetes) or antidiabetic medication use (oral hypoglycemics or insulin) during the prior year No evidence of pregnancy or bariatric surgery during the prior year	Inclusion: At least 1 year of continuous enrollment in medical and pharmacy claims Exclusion: Diabetes, pregnancy, and bariatric surgery defined using diagnosis and procedure codes
Baseline	Randomization would occur once all eligibility criteria are met	Treatment comparison would start on the date of treatment initiation once all eligibility criteria are met; anticonvulsant use defined using dispensing data
Treatment strategies	Patients would be randomized to initiate 1 of 4 treatment strategies: carbamazepine lamotrigine oxcarbazepine valproate Treatment adherence defined as daily adherence to assigned treatment	Date of medication initiation was the date of the first prescription filled Treatment adherence assessed using treatment diary, allowing a 100% grace period
Treatment assignment	Randomly assigned to a treatment strategy at baseline	Treatment not assigned randomly, and therefore requires confounding adjustment
Outcome	Type 2 diabetes	Same as target trial Defined using a computable phenotype of type 2 diabetes
Follow-up period	Follow patients for 5 years or until the onset of type 2 diabetes or trial disenrollment	Follow patients for 5 years or until the onset of type 2 diabetes, end of continuous enrollment in the medical and pharmacy claims, or the end of available data
Contrast of interest	Intention-to-treat effect Per-protocol effect	Observational analog

^a^ Unless otherwise stated, all specifications were the same for the pediatric and adult target trials.

#### Eligibility Criteria, Time 0

In the adult trial, eligible adults planning to initiate an anticonvulsant mood stabilizer would be aged 20 to 65 years and have continuous enrollment in a health care plan over the prior year. Patients would be excluded if they had previously used an anticonvulsant medication, had prior diagnoses of diabetes (type 1, type 2, secondary, or gestational diabetes) or antidiabetic medication use except metformin (ie, patients treated with metformin for a condition besides diabetes would be eligible for inclusion), or had a pregnancy or bariatric surgery in the previous year. Baseline (time 0) would be defined as the first time all eligibility criteria were met. For the pediatric trial, the eligibility criteria would be the same as the adult trial, except patients would be aged 10 to 19 years.

#### Treatment Strategies, Assignment

Patients would be randomized at baseline to initiate 1 of the following medications: (1) carbamazepine, (2) lamotrigine, (3) oxcarbazepine, or (4) valproate. Treatment adherence over follow-up would be defined as daily adherence to the assigned medication and no receipt of a different anticonvulsant medication.

#### Follow-up Period and Estimated Outcomes

Patients would be followed for 5 years or until the onset of T2D or trial disenrollment. The outcome would be incident T2D during follow up. The target trial would estimate the outcomes of treatment initiation on incident T2D (intention-to-treat [ITT] effect) and the outcomes of adherence to assigned treatment strategy (per-protocol [PP] effect). Lamotrigine would serve as the reference group because it is not associated with weight gain.^23,24,25,26^

### Emulation of Target Trial Using Health Care Utilization Data

After specifying the target trial, we emulated the components within the IBM MarketScan Commercial Database (2010-2019), comprising more than 200 million patients, most of whom are covered by employer-sponsored insurance. This database captures longitudinal patient-level health care utilization data, including demographics, inpatient and outpatient medical diagnoses and procedures, and outpatient medication dispensings.

#### Eligibility Criteria, Time 0

Individuals were selected to match the eligibility criteria of the target trial as closely as possible. We required 1 or more years of continuous enrollment in the medical and pharmacy claims and defined eligibility criteria using outpatient dispensings, diagnoses, and procedure codes (definitions in eTable 1 in the Supplement). The treatment comparison started on the date of treatment initiation (time 0) once all eligibility criteria were met.

#### Treatment Strategies, Assignment

Treatment strategies were classified according to the medication dispensed at baseline using the first observed dispensing (classifications defined in eTable 2 in the Supplement). Treatment adherence was assessed using a treatment diary constructed by dispensing dates and days supply for each medication. We allowed a gap between dispensings of up to twice the days supply of the previous dispensing (100% grace period; eg, for a 30-day dispensing, we allowed an additional 30 days between the end of supply and the next prescription filled).^37^

Since patients were not randomized to a treatment strategy, treatment initiation may be associated with characteristics that are also associated with T2D (baseline confounders). Therefore, we adjusted for 50 or more baseline covariates that were potential confounders or proxies of confounders: demographics, treatment indications, metabolic conditions, metabolic laboratory tests ordered, psychiatric conditions, lifestyle factors (eg, smoking and drug abuse), dispensings for other psychiatric medications, dispensings for other medications associated with weight change, health care utilization, and a comorbidity index (eTable 3 in the Supplement).^38,39^ Covariates were measured during the year prior to treatment initiation.

Additionally, treatment adherence over follow-up may also be associated with time-varying factors associated with T2D. To account for potential selection bias due to treatment discontinuation or treatment switching, we adjusted for the previously described baseline covariates, as well as measured time-varying covariates: diagnoses and medications associated with weight change or T2D, treatment indications, health care utilization, and lifestyle factors (eTable 3 in the Supplement).

#### Follow-up Period and Estimated Outcome

We followed patients for up to 5 years or until loss to follow-up, defined as the end of continuous enrollment or end of available data. We defined incident T2D during follow-up using previously validated algorithms based on diagnosis codes and antidiabetic medications (positive predictive value, 90% for adults^40^ and 87% for children; negative predictive value, 96%^41^). The contrasts of interest were the observational analog to those defined for the target trial.

### Statistical Analysis

To account for baseline confounding, we applied inverse probability of treatment weights. We used multinomial logistic regression to estimate stabilized treatment weights. To account for potential selection bias, we applied inverse probability of censoring weights.^42^ In the ITT analysis, these weights were estimated on the basis of baseline covariates. In the PP analysis, inverse probability of censoring weights were estimated according to baseline and time-varying covariates to account for potential selection bias due to loss to follow-up, treatment discontinuation, or treatment switching (details in eAppendix in the Supplement).

We calculated the ITT and PP estimates as contrasts in the 2-year and 5-year absolute risks of T2D for each initiation and adherence intervention, respectively. We estimated absolute risks using pooled logistic regression weighted by the product of inverse probability of treatment weights and inverse probability of censoring weights. We truncated weights at the first and 99th percentiles.^43^ We used nonparametric bootstrapping to estimate 95% CIs (details in eAppendix in the Supplement). To explore potential effect modification, we conducted exploratory subgroup analyses for age at treatment initiation and major treatment indications (eg, bipolar disorder or epilepsy).

We conducted several sensitivity analyses to evaluate the robustness of our findings. First, we quantified the outcomes of potential unmeasured confounding using the e-value.^44,45^ Second, to examine the bias-variance trade-off associated with weight truncation,^43^ we implemented different ways to truncate inverse probability weights. Third, we relaxed our definition of adherence in the PP analysis by expanding the allowable gap between dispensings to twice the days supplied of the current dispensing (ie, 200% grace period).

In these subgroup and sensitivity analyses, we used pooled logistic regression to estimate a hazard ratio and the sandwich variance estimator to compute 95% CIs.^46,47^ These hazard ratios over the 5-year follow-up may be uninformative when the hazard ratio changes over time.^48^ The purpose of using a single value was to provide a concise summary of the overall extent to which these analyses differed from our primary analysis. Data were analyzed using SAS statistical software version 9.4 (SAS Institute) from August 2020 to May 2021.

## Results

### Patient Characteristics

The adult trial emulation consisted of 274 206 adults (159 428 women [58%]; mean [SD] age, 39.9 [13.2] years) who initiated an anticonvulsant mood stabilizer. There were 26 641 carbamazepine initiators, 132 739 lamotrigine initiators, 24 226 oxcarbazepine initiators, and 90 600 valproate initiators (eFigure 1 in the Supplement). Compared with initiators of other mood stabilizers, initiators of carbamazepine were the least likely to have a diagnosis of bipolar disorder (13.8% of carbamazepine initiators vs 34.9% to 38.7% among other treatment groups) and the most likely to have a diagnosis of neuropathic pain (40.8% vs 10.5% to 20.3%) (Table 2 and eTable 4 in the Supplement). Psychiatric conditions and medications were the least prevalent among initiators of carbamazepine (eg, 6416 carbamazepine initiators [24.1%] had a diagnosis of depression vs 77 693 lamotrigine initiators [58.5%]). The presence of diagnostic codes for obesity and metabolic conditions were similar across treatment groups.

**Table 2.** Baseline Characteristics of Adults Aged 20 Years and Older With Anticonvulsant Mood Stabilizer Treatment in MarketScan, 2010-2019

Characteristic	Patients, No. (%)							
	Unadjusted				Weighted by inverse probability of treatment			
	Carbamazepine (n = 26 641)	Lamotrigine (n = 132 739)	Oxcarbazepine (n = 24 226)	Valproate (n = 90 600)	Carbamazepine (n = 26 805)	Lamotrigine (n = 130 670)	Oxcarbazepine (n = 24 712)	Valproate (n = 89 831)
Demographics								
Age, mean (SD), y	45.5 (12.5)	38.0 (12.8)	39.5 (13.4)	41.0 (13.3)	39.5 (13.1)	38.0 (12.8)	39.8 (13.5)	39.8 (13.1)
Female	16 069 (60.3)	87 039 (65.6)	14 418 (59.5)	41 902 (46.2)	15 123 (56.4)	75 729 (58.0)	14 417 (58.3)	51 569 (57.4)
Male	10 572 (39.7)	45 700 (34.4)	9808 (40.5)	48 698 (53.8)	11 682 (43.6)	54 941 (42.0)	10 295 (41.7)	38 262 (42.6)
Combined comorbidity index, mean (SD)	0.5 (1.2)	1.0 (1.0)	0.9 (1.2)	0.9 (1.3)	1.0 (1.3)	1.0 (1.0)	0.9 (1.2)	0.9 (1.2)
Medical diagnoses								
Bipolar disorder	3669 (13.8)	51 393 (38.7)	8445 (34.9)	32 447 (35.8)	10 829 (40.4)	48 095 (36.8)	8973 (36.3)	33 303 (37.1)
Epilepsy or convulsions	2156 (8.1)	5715 (4.3)	1680 (6.9)	9458 (10.4)	2329 (8.7)	9649 (7.4)	1763 (7.1)	6393 (7.1)
Migraine or headache	6917 (26.0)	19 657 (14.8)	4298 (17.7)	25 769 (28.4)	6083 (22.7)	26 740 (20.5)	5205 (21.1)	18 972 (21.1)
Neuropathic pain	10 876 (40.8)	13 913 (10.5)	4913 (20.3)	10 156 (11.2)	3596 (13.4)	18 084 (13.8)	3819 (15.5)	12 792 (14.2)
Obesity or overweight	2931 (11.0)	15 097 (11.4)	2971 (12.3)	8258 (9.1)	2923 (10.9)	14 290 (10.9)	2660 (10.8)	9816 (10.9)
Weight management	313 (1.2)	2142 (1.6)	335 (1.4)	861 (1.0)	341 (1.3)	1767 (1.4)	325 (1.3)	1176 (1.3)
Prediabetes	1251 (4.7)	5096 (3.8)	1031 (4.3)	3556 (3.9)	1209 (4.5)	5284 (4.0)	993 (4.0)	3648 (4.1)
Anxiety	5868 (22.0)	61 497 (46.3)	9560 (39.5)	27 069 (29.9)	10 898 (40.7)	51 181 (39.2)	9602 (38.9)	35 189 (39.2)
Depression	6416 (24.1)	77 693 (58.5)	11 144 (46)	33 280 (36.7)	13 280 (49.5)	63 468 (48.6)	11 824 (47.8)	43 411 (48.3)
Psychotic disorders	915 (3.4)	5403 (4.1)	1745 (7.2)	9770 (10.8)	2274 (8.5)	8904 (6.8)	1712 (6.9)	6077 (6.8)
Medications								
Lithium	630 (2.4)	6706 (5.1)	1047 (4.3)	4048 (4.5)	1689 (6.3)	6605 (5.1)	1206 (4.9)	4614 (5.1)
Antipsychotics	3121 (11.7)	35 639 (26.8)	6671 (27.5)	26 468 (29.2)	8547 (31.9)	35 833 (27.4)	6727 (27.2)	24 655 (27.4)
Antidepressants	8318 (31.2)	80 521 (60.7)	12 359 (51.0)	39 202 (43.3)	14 684 (54.8)	68 947 (52.8)	12 817 (51.9)	47 217 (52.6)
Stimulants	2152 (8.1)	24 058 (18.1)	3886 (16.0)	10 111 (11.2)	4453 (16.6)	19 876 (15.2)	3715 (15.0)	13 722 (15.3)
Oral corticosteroids	7161 (26.9)	24 830 (18.7)	5465 (22.6)	18 346 (20.2)	5664 (21.1)	26 295 (20.1)	5047 (20.4)	18 370 (20.4)
Health care utilization								
Outpatient visits, median (IQR), No.	9 (5-18)	12 (6-23)	11 (6-21)	10 (5-19)	12 (6-23)	12 (6-22)	11 (6-21)	11 (5-21)
MH outpatient visits, median (IQR), No.	0 (0-2)	4 (1-10)	3 (0-8)	2 (0-6)	3 (0-9)	3 (1-9)	3 (0-8)	3 (0-8)
Distinct generic drugs, median (IQR), No.	7 (3-11)	7 (4-11)	7 (4-12)	7 (4-12)	8 (4-12)	7 (4-12)	7 (4-11)	7 (4-11)
Any hospitalization	4099 (15.4)	17 392 (13.1)	5302 (21.9)	20 799 (22.9)	6178 (23)	23 780 (18.2)	4357 (17.6)	16 436 (18.3)
Any MH hospitalization	2456 (9.2)	11 835 (8.9)	3834 (15.8)	13 949 (15.4)	4299 (16)	16 171 (12.4)	2934 (11.9)	11 137 (12.4)

Abbreviation: MH, mental health.

The pediatric trial emulation consisted of 74 005 children (38 672 girls [52%]; mean [SD] age, 15.6 [2.6] years), which included 2532 carbamazepine initiators, 36 394 lamotrigine initiators, 12 434 oxcarbazepine initiators, and 22 645 valproate initiators (eFigure 1 in the Supplement). The distributions of potential treatment indications were generally similar across treatment groups (Table 3 and eTable 5 in the Supplement). Bipolar disorder was the most common potential indication (range, 4556 oxcarbazepine initiators [36.6% ] to 14 723 lamotrigine initiators [40.5%]). Compared with the other treatment groups, lamotrigine initiators were more likely to have a psychiatric diagnosis. The distributions of nonpsychiatric diagnoses and medications, including codes for obesity and metabolic conditions, were similar across treatment groups. In both adult and pediatric trial emulations, patient characteristics were similar across treatment groups after weighting by inverse probability of treatment weights (Table 2 and Table 3 and eTable 6 and eTable 7 in the Supplement).

**Table 3.** Baseline Characteristics of Children Aged 10 to 19 Years With Anticonvulsant Mood Stabilizer Treatment in MarketScan, 2010-2019

Characteristic	Patients, No. (%)							
	Unadjusted				Weighted by Inverse Probability of Treatment			
	Carbamazepine (n = 2532)	Lamotrigine (n = 36 394)	Oxcarbazepine (n = 12 434)	Valproate (n = 22 645)	Carbamazepine (n = 2548)	Lamotrigine (n = 36 136)	Oxcarbazepine (n = 12 495)	Valproate (n = 21 922)
Demographics								
Age, mean (SD), y	15.6 (2.7)	16.0 (2.4)	14.7 (2.7)	15.5 (2.7)	15.7 (2.7)	15.6 (2.6)	15.6 (2.6)	15.6 (2.6)
Female	1193 (47.1)	24 273 (66.7)	6023 (48.4)	7183 (31.7)	1320 (51.8)	18 975 (52.5)	6627 (53.0)	11 058 (50.4)
Male	1339 (52.9)	12 121 (33.3)	6411 (51.6)	15 462 (68.3)	1228 (48.2)	17 161 (47.5)	5868 (47.0)	10 864 (49.6)
Pediatric comorbidity index, mean (SD)	6.0 (4.3)	6.5 (3.8)	6.2 (3.9)	5.8 (4.1)	6.4 (4.2)	6.3 (4.0)	6.3 (4.0)	6.3 (4.0)
Medical diagnoses								
Bipolar disorder	968 (38.2)	14 723 (40.5)	4556 (36.6)	8840 (39.0)	1073 (42.1)	14 461 (40.0)	5110 (40.9)	8960 (40.9)
Epilepsy or convulsions	344 (13.6)	2204 (6.1)	1862 (15.0)	3627 (16.0)	277 (10.9)	3838 (10.6)	1310 (10.5)	2514 (11.5)
Migraine or headache	415 (16.4)	4679 (12.9)	1403 (11.3)	4835 (21.4)	378 (14.8)	5535 (15.3)	1904 (15.2)	3539 (16.1)
Neuropathic pain	176 (7.0)	1155 (3.2)	279 (2.2)	569 (2.5)	79 (3.1)	1070 (3.0)	388 (3.1)	644 (2.9)
Obesity or overweight	161 (6.4)	2649 (7.3)	862 (6.9)	1117 (4.9)	160 (6.3)	2377 (6.6)	834 (6.7)	1387 (6.3)
Weight management	62 (2.4)	1488 (4.1)	498 (4.0)	669 (3.0)	85 (3.3)	1318 (3.6)	454 (3.6)	745 (3.4)
Prediabetes	22 (0.9)	315 (0.9)	92 (0.7)	175 (0.8)	22 (0.9)	300 (0.8)	95 (0.8)	188 (0.9)
Anxiety	714 (28.2)	17 075 (46.9)	4235 (34.1)	5976 (26.4)	969 (38.0)	13 810 (38.2)	4846 (38.8)	8048 (36.7)
Depression	975 (38.5)	22 100 (60.7)	5477 (44.0)	8632 (18.7)	1289 (50.6)	18 137 (50.2)	6352 (50.8)	10 715 (48.9)
Psychotic disorders	257 (10.2)	2694 (7.4)	1092 (8.8)	2755 (12.2)	241 (9.4)	3347 (9.3)	1169 (9.4)	2078 (9.5)
Medications								
Lithium	117 (4.6)	1519 (4.2)	332 (2.7)	847 (3.7)	114 (4.5)	1460 (4.0)	493 (3.9)	954 (4.4)
Antipsychotics	913 (36.1)	12 898 (35.4)	4527 (36.4)	9089 (40.1)	998 (39.1)	13 727 (38.0)	4818 (38.6)	8435 (38.5)
Antidepressants	1001 (39.5)	21 777 (59.8)	5760 (46.3)	8272 (36.5)	1297 (50.9)	18 251 (50.5)	6360 (50.9)	10 819 (49.4)
Stimulants	807 (31.9)	11 306 (31.1)	4793 (38.5)	8010 (35.4)	878 (34.4)	12 355 (34.2)	4306 (34.5)	7551 (34.4)
Oral corticosteroids	329 (13.0)	4210 (11.6)	1319 (10.6)	2706 (11.9)	304 (11.9)	4170 (11.5)	1463 (11.7)	2545 (11.6)
Health care utilization								
Outpatient visits, median (IQR), No.	9 (6-19)	13 (7-26)	11 (5-21)	9 (5-18)	11 (6-23)	12 (6-23)	12 (6-23)	11 (5-22)
MH outpatient visits, median (IQR), No.	3 (0-9)	6 (2-17)	4 (1-12)	3 (0-10)	5 (1-14)	5 (1-14)	5 (1-14)	4 (1-12)
Distinct generic drugs, median (IQR), No	5 (2-8)	5 (3-8)	4 (2-7)	5 (3-8)	5 (3-8)	5 (3-8)	5 (3-8)	5 (3-8)
Any hospitalization	799 (31.6)	8884 (24.4)	3682 (29.6)	6652 (29.4)	703 (27.6)	9651 (26.7)	3455 (27.7)	6023 (27.5)
Any MH hospitalization	681 (26.9)	8074 (22.2)	3096 (24.9)	5528 (24.4)	614 (24.1)	8457 (23.4)	3017 (24.1)	5191 (23.7)

Abbreviation: MH, mental health.

### Adults

In the ITT analysis, 8432 patients (3.1%) developed T2D over follow-up (mean [SD], 1.9 [1.5] years). The crude incidence rate ranged from 14.2 T2D cases per 1000 person-years among lamotrigine initiators to 19.2 T2D cases per 1000 person-years among valproate initiators (eTable 8 in the Supplement). After adjusting for baseline confounding, the 5-year T2D risk difference (RD) comparing initiation of valproate to initiation of lamotrigine was 1.17% (95% CI, 0.66 to 1.76). This corresponded to a number needed to harm of 87 patients initiating valproate for 1 patient to develop T2D within 5 years. The magnitude of the RD was smaller for initiation of carbamazepine (5-year RD, 0.49%; 95% CI, −0.57% to 1.51%) and oxcarbazepine (5-year RD, 0.27%; 95% CI, −0.47% to 0.96%) compared with initiation of lamotrigine (Figure, Table 4 and eTable 9 in the Supplement).

**Figure.**
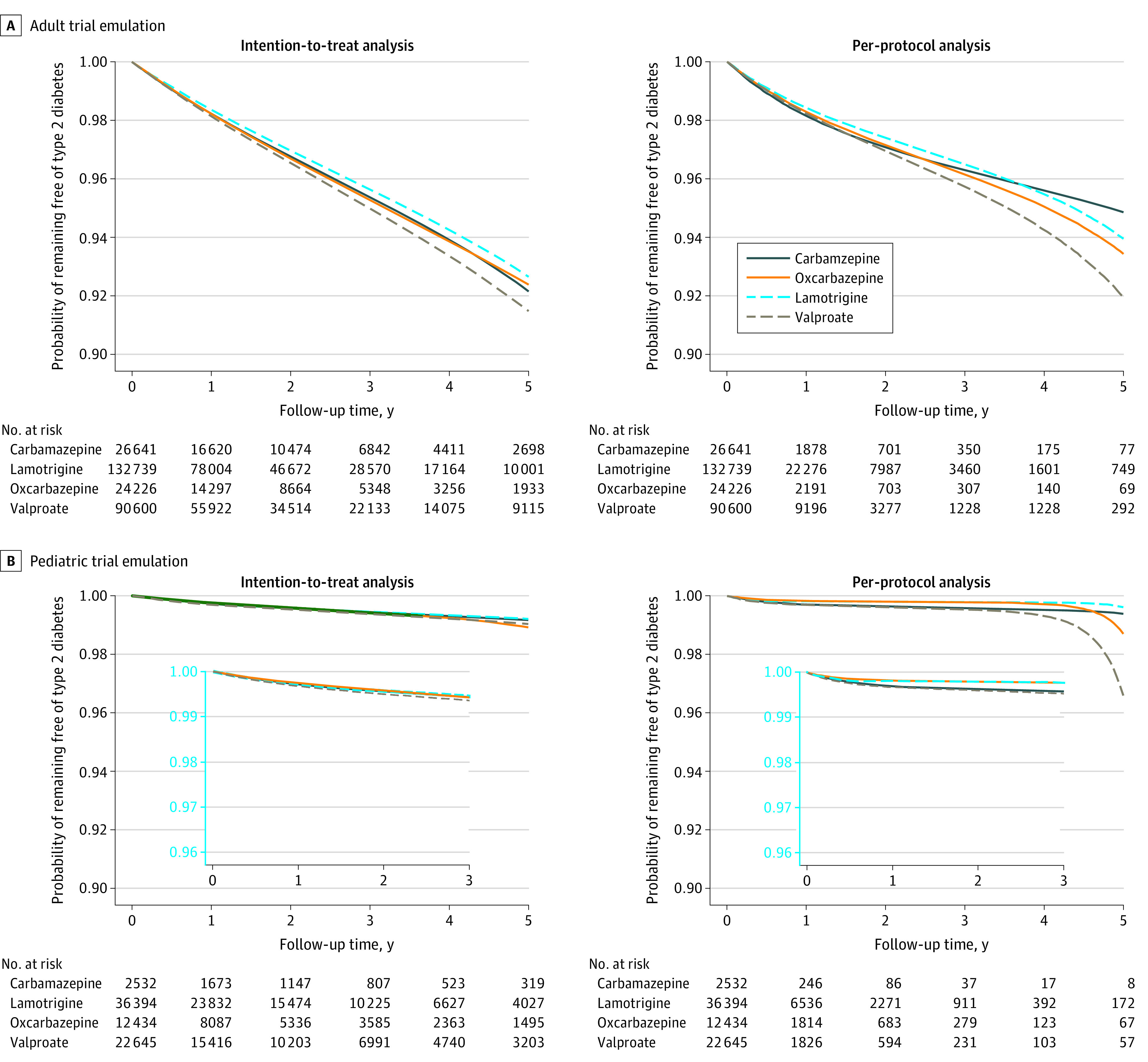
Weighted Survival Curves Comparing Anticonvulsant Mood Stabilizer Treatment Adjusted survival curves were weighted by the inverse probability of treatment and the inverse probability of censoring (adjusted for baseline covariates in the intention-to-treat analysis, adjusted for baseline and time-varying covariates and standardized to the joint distribution of a subset of baseline covariates in the per-protocol analysis). Findings from the pediatric per-protocol analysis should be interpreted in the context of its few T2D events and short mean follow-up. Inset charts in Panel B show a zoomed-in version of the survival curves. Effect estimates and 95% CIs are highlighted in Table 3.

**Table 4.** Adjusted Risk Differences Comparing the Incidence of T2D Across Mood Stabilizer Treatment

Treatment group	Adjusted risk difference, % (95% CI)^a^	
	2-y	5-y
Adult trial		
Intention-to-treat		
Carbamazepine	0.21 (−0.14 to 0.61)	0.49 (−0.57 to 1.51)
Lamotrigine	1 [Reference]	1 [Reference]
Oxcarbazepine	0.28 (−0.03 to 0.59)	0.27 (−0.47 to 0.96)
Valproate	0.43 (0.24 to 0.67)	1.17 (0.66 to 1.76)
Per-protocol		
Carbamazepine	0.32 (−0.41 to 1.26)	−0.90 (−3.72 to 3.30)
Lamotrigine	1 [Reference]	1 [Reference]
Oxcarbazepine	0.24 (−0.47 to 0.93)	0.51 (−1.89 to 3.72)
Valproate	0.45 (0.04 to 0.91)	1.99 (−0.64 to 5.31)
Pediatric trial		
Intention-to-treat		
Carbamazepine	0.01 (−0.25 to 0.29)	0.04 (−0.42 to 0.64)
Lamotrigine	1 [Reference]	1 [Reference]
Oxcarbazepine	−0.02 (−0.18 to 0.15)	0.29 (−0.12 to 0.69)
Valproate	0.06 (−0.10 to 0.22)	0.18 (−0.09 to 0.49)
Per-protocol		
Carbamazepine	0.16 (−0.15 to 0.66)	0.22 (−0.20 to 7.04)
Lamotrigine	1 [Refeference]	1 [Reference]
Oxcarbazepine	−0.01 (−0.14 to 0.17)	0.92 (−0.14 to 3.11)
Valproate	0.19 (−0.06 to 0.60)	3.06 (0.16 to 9.29)

^a^ Adjusted risk differences were weighted by the inverse probability of treatment and the inverse probability of censoring (adjusted for baseline covariates in the intention-to-treat analysis, adjusted for baseline and time-varying covariates in the per-protocol analysis).

The observed follow-up was substantially shorter in the PP analysis because of additional censoring upon protocol nonadherence (mean [SD], duration of adherence, 6.0 [8.0] months) (eTable 8 in the Supplement). Consistent with the ITT analysis, valproate use had the highest incidence rate of T2D compared with lamotrigine use after adjustment (5-year RD, 1.99%; 95% CI, −0.64% to 5.31%) (Figure, Table 4, and eTable 9 in the Supplement).

### Children

In the ITT analysis, 333 patients (0.5%) developed T2D (mean [SD] follow-up, 2.1 [1.6] years). The crude incidence rate ranged from 2.1 T2D cases per 1000 person-years among oxcarbazepine initiators to 2.5 T2D cases per 1000 person-years among carbamazepine initiators (eTable 8 in the Supplement). Compared with initiation of lamotrigine, the confounding-adjusted 5-year risk of T2D was 0.29% (95% CI, −0.12% to 0.69%) higher for initiation of oxcarbazepine and 0.18% (95% CI, −0.09% to 0.49%) higher for initiation of valproate; the 5-year RD of T2D was nearly 0 for initiation of carbamazepine (Figure, Table 4, and eTable 9 in the Supplement).

In the PP analysis, there were even fewer T2D events (110 events) and substantially shorter follow-up time (mean [SD], 6.2 [8.4] months) (eTable 8 in the Supplement). Therefore, we were unable to detect meaningful differences in T2D risk across treatment groups (Figure, Table 4, and eTable 9 in the Supplement).

### Subgroup Analyses

In subgroup analyses for adults, findings were generally consistent across age and treatment indication (eTable 10 in the Supplement). Subgroup analyses were not conducted in children given the low event rate.

### Sensitivity Analyses

We quantified the potential role of unmeasured confounding on observed point estimates in the ITT analysis in eFigure 2 in the Supplement and found that the magnitude of unmeasured confounding may not be strong enough to explain away our findings. Next, we found that applying different ways to truncate inverse probability weights did not meaningfully alter estimates (eTable 11 in the Supplement). Finally, extending the allowable gap between prescriptions in the PP analysis resulted in slightly longer follow-up (mean [SD], 7.4 [9.3] months among adults and 8.0 [10.0] months among children), but did not alter estimates (eTable 12 in the Supplement).

## Discussion

In this cohort study, we estimated that initiation of and adherence to valproate was associated with the highest risk of developing T2D, with a number needed to harm of 87 patients initiating valproate for 1 patient to develop T2D within 5 years compared with initiation of lamotrigine. Of the 4 medications examined, lamotrigine treatment was associated with the lowest risk of developing T2D. These findings highlight that the choice of which anticonvulsant mood stabilizer to initiate may have meaningful associations with the incidence of T2D. Findings were generally consistent across several subgroup and sensitivity analyses. The comparative safety of mood stabilizer treatment was generally in the same direction for pediatric patients, where we observed the highest risk of T2D for initiation of valproate and oxcarbazepine. However, the estimates were small and imprecise. Nevertheless, if the potential risk of T2D during childhood is confirmed in larger studies, this small difference could be meaningful. Youth-onset T2D is generally a more aggressive condition than adult-onset T2D, and children will have to live most of their lives with T2D.^49^ In both children and adults, we found that initiation of lamotrigine was associated with the lowest risk of developing T2D. These findings highlight that across all age groups, patients and clinicians concerned about the potential risk of developing T2D could consider initiating lamotrigine if effectiveness of treatment for the condition in which it is indicated is similar and the reduced metabolic risk outweighs the other known risks associated with lamotrigine treatment.^50,51^

Previous studies,^27,28,29,30^ which were mostly small studies and case series, have reported a potential association between valproate and metabolic disorders. In our nationwide cohort study, we confirmed that initiating valproate is associated with an increased risk of developing T2D in adults. We also expand the available evidence by evaluating the risk of T2D for other anticonvulsant mood stabilizers, which were generally associated with a lower risk of T2D than valproate.

Emulating target trials within health care databases can fill gaps in evidence on medication safety in a more rapid and less costly manner than conducting RCTs. Compared with RCTs, our study had a larger sample size that provided the opportunity to examine individual mood stabilizer medications and capture a rare end point. We sought to obtain the estimates that we would have obtained if we had been able to conduct these target trials. Although our observational study does not reflect a true replication of RCTs (and RCTs have barriers to valid causal inferences, including imperfect protocol adherence and loss to follow-up), application of the target trial framework helped avoid common methodological pitfalls.^36^

### Limitations

There are limitations to our study. First, the lack of randomization may result in confounded estimates. Although some factors associated with the risk of T2D are unmeasured or poorly captured in claims data (eg, obesity, body mass index, and blood glucose levels), they are unlikely to be associated with the choice of mood stabilizer treatment. Therefore, the magnitude of such unmeasured confounding would likely not be strong enough to explain away our findings, and the plausible scenarios are quantified in eFigure 2 in the Supplement. Second, poor adherence to treatment resulted in a short follow-up period in the PP analysis. This limited our ability to capture T2D events. The follow-up time remained short even after extending the allowable gap between dispensings, highlighting that actual adherence to mood stabilizer treatment is poor. Third, exposure, outcome, or covariate misclassification cannot be excluded, as claims databases do not include laboratory data or weight measurements. Exposure was defined according to medication dispensings, which may not indicate use. To explore the potential for exposure misclassification, we required continuous treatment in the PP analysis and considered a different definition for treatment discontinuation in sensitivity analyses. To minimize outcome misclassification, we used validated, age-specific definitions of T2D that had high positive predictive values in health care databases.^40,41^ Although this study was intended to include patients without T2D at baseline, it is possible misclassification of the eligibility criteria resulted in the inclusion of some patients with impaired blood glucose at baseline. Additionally, findings may differ in other populations, such as publicly insured patients.

## Conclusions

This study, which emulated the approach of a target trial using observational data, found that valproate use in adults was associated with an increased risk of developing T2D compared with lamotrigine use. Although the comparative safety of mood stabilizer treatment was generally in the same direction for children, RDs were small and underpowered. These findings highlight that at the population level, the choice of which anticonvulsant mood stabilizer to initiate could have meaningful associations with the incidence of T2D. Patients and clinicians concerned about the potential metabolic adverse effects of treatment should consider initiating lamotrigine, which was associated with the lowest risk of T2D. In the absence of RCTs, observational studies that emulate target trials can generate the age-specific drug safety data needed to inform treatment decision-making.

## Notes

Supplement. **eTable 1.** Details of Eligibility Criteria **eTable 2.** Study Definitions for Treatment Strategies **eTable 3.** List of Baseline and Time-Varying Covariates **eAppendix.** Supplemental Methods: Details on Estimating Inverse Probability Weights and Treatment Effects **eFigure 1.** Flow Diagram of Cohort Assembly **eTable 4.** Unadjusted Baseline Characteristics of Adults Who Initiated Anticonvulsant Mood Stabilizer Treatment in MarketScan **eTable 5.** Unadjusted Baseline Characteristics of Children Who Initiated Anticonvulsant Mood Stabilizer Treatment in MarketScan **eTable 6.** Baseline Characteristics of Adults Who Initiated Anticonvulsant Mood Stabilizer Treatment in MarketScan After Accounting for Baseline Confounding **eTable 7.** Baseline Characteristics of Children Who Initiated Anticonvulsant Mood Stabilizer Treatment in MarketScan After Accounting for Baseline Confounding **eTable 8.** Absolute Rate of Type 2 Diabetes Among Patients Within the Adult and Pediatric Trial Emulation **eTable 9.** Hazard Ratios and 95% Confidence Intervals Comparing the Incidence of Type 2 Diabetes Across Mood Stabilizer Treatment, By Level of Adjustment **eTable 10.** Adjusted Hazard Ratios and 95% Confidence Intervals Comparing the Incidence of Type 2 Diabetes Across Mood Stabilizer Treatment in Adults, Stratified by Age and Treatment Indication **eFigure 2.** Sensitivity Analysis Evaluating the Potential Role of Unmeasured Confounding on Observed Point Estimates in the Intention-to-Treat Analysis **eTable 11.** Sensitivity Analysis Evaluating the Potential Impact of Truncating Inverse Probability Weights **eTable 12.** Sensitivity Analysis Evaluating the Potential Role of Different Grace Periods in the Per-Protocol Analysis
eReferences
