# Supplementary material for: Comparison of Rates of Type 2 Diabetes in Adults and Children Treated With Anticonvulsant Mood Stabilizers
Source: JAMA Netw Open. 2022 Apr 6;5(4):e226484. doi: 10.1001/jamanetworkopen.2022.6484 (PMC8987905; doi:10.1001/jamanetworkopen.2022.6484)
Supplement: Supplement. — eTable 1. Details of Eligibility Criteria eTable 2. Study Definitions for Treatment Strategies eTable 3. List of Baseline and Time-Varying Covariates eAppendix. Supplemental Methods: Details on Estimating Inverse Probability Weights and Treatment Effects eFigure 1. Flow Diagram of Cohort Assembly eTable 4. Unadjusted Baseline Characteristics of Adults Who Initiated Anticonvulsant Mood Stabilizer Treatment in MarketScan eTable 5. Unadjusted Baseline Characteristics of Children Who Initiated Anticonvulsant Mood Stabilizer Treatment in MarketScan eTable 6. Baseline Characteristics of Adults Who Initiated Anticonvulsant Mood Stabilizer Treatment in MarketScan After Accounting for Baseline Confounding eTable 7. Baseline Characteristics of Children Who Initiated Anticonvulsant Mood Stabilizer Treatment in MarketScan After Accounting for Baseline Confounding eTable 8. Absolute Rate of Type 2 Diabetes Among Patients Within the Adult and Pediatric Trial Emulation eTable 9. Hazard Ratios and 95% Confidence Intervals Comparing the Incidence of Type 2 Diabetes Across Mood Stabilizer Treatment, By Level of Adjustment eTable 10. Adjusted Hazard Ratios and 95% Confidence Intervals Comparing the Incidence of Type 2 Diabetes Across Mood Stabilizer Treatment in Adults, Stratified by Age and Treatment Indication eFigure 2. Sensitivity Analysis Evaluating the Potential Role of Unmeasured Confounding on Observed Point Estimates in the Intention-to-Treat Analysis eTable 11. Sensitivity Analysis Evaluating the Potential Impact of Truncating Inverse Probability Weights eTable 12. Sensitivity Analysis Evaluating the Potential Role of Different Grace Periods in the Per-Protocol Analysis eReferences [file jamanetwopen-e226484-s001.pdf]

Supplemental Online Content

Sun JW, Young JG, Sarvet AL, et al. Comparison of rates of type 2 diabetes in adults and children treated with anticonvulsant mood stabilizers. *JAMA Netw Open*. 2022;5(4):e226484. doi:10.1001/jamanetworkopen.2022.6484

eTable 1. Details of Eligibility Criteria
eTable 2. Study Definitions for Treatment Strategies
eTable 3. List of Baseline and Time-Varying Covariates
eAppendix. Supplemental Methods: Details on Estimating Inverse Probability Weights and Treatment Effects
eFigure 1. Flow Diagram of Cohort Assembly
eTable 4. Unadjusted Baseline Characteristics of Adults Who Initiated Anticonvulsant Mood Stabilizer Treatment in MarketScan
eTable 5. Unadjusted Baseline Characteristics of Children Who Initiated Anticonvulsant Mood Stabilizer Treatment in MarketScan
eTable 6. Baseline Characteristics of Adults Who Initiated Anticonvulsant Mood Stabilizer Treatment in MarketScan After Accounting for Baseline Confounding
eTable 7. Baseline Characteristics of Children Who Initiated Anticonvulsant Mood Stabilizer Treatment in MarketScan After Accounting for Baseline Confounding
eTable 8. Absolute Rate of Type 2 Diabetes Among Patients Within the Adult and Pediatric Trial Emulation
eTable 9. Hazard Ratios and 95% Confidence Intervals Comparing the Incidence of Type 2 Diabetes Across Mood Stabilizer Treatment, By Level of Adjustment
eTable 10. Adjusted Hazard Ratios and 95% Confidence Intervals Comparing the Incidence of Type 2 Diabetes Across Mood Stabilizer Treatment in Adults, Stratified by Age and Treatment Indication
eFigure 2. Sensitivity Analysis Evaluating the Potential Role of Unmeasured Confounding on Observed Point Estimates in the Intention-to-Treat Analysis
eTable 11. Sensitivity Analysis Evaluating the Potential Impact of Truncating Inverse Probability Weights
eTable 12. Sensitivity Analysis Evaluating the Potential Role of Different Grace Periods in the Per-Protocol Analysis
eReferences

This supplemental material has been provided by the authors to give readers additional information about their work.

eTable 1. Details of Eligibility Criteria

Eligibility Criteria	Implementation in MarketScan	Rationale
Inclusion Criteria		
Age 20-65 years for adult trial<br>Age 10-19 years for pediatric trial	Age assessed on the date of treatment initiation	Both children and adults were included because patients are treated with anticonvulsant mood stabilizers across the life course
Continuous enrollment in a health plan for at least 1 year	Require continuous pharmacy and medical enrollment during the year prior to treatment initiation	To ensure complete capture of the claims data
Exclusion Criteria		
No anticonvulsant medication use (including those not used as mood stabilizers) during the prior year	Exclude patients with a dispensing of any anticonvulsant mood stabilizer in the year prior to treatment initiation	New users only
No diagnosis of diabetes (type 1, type 2, secondary or gestational diabetes) or antidiabetic medication use (oral hypoglycemics or insulin) during the prior year	Exclude patients with a diagnosis of diabetes (type 1, type 2, secondary or gestational diabetes) or dispensing of an antidiabetic medication (except metformin*) during the year prior to treatment initiation<br><br>*Since metformin is used to treat conditions besides diabetes, patients who were treated with metformin but were not diagnosed with diabetes were eligible for inclusion in the study	Exclude patients with the outcome at baseline; Minimize reverse causation
No evidence of pregnancy during the prior year	Exclude patients with a diagnosis or procedure code suggesting pregnancy or delivery of infant in the year prior to treatment initiation	Treatment decisions differ for pregnant women
No evidence of bariatric surgery during the prior year	Exclude patients with a procedure code for bariatric surgery in the year prior to treatment initiation	Patterns of weight gain and metabolic risk differ substantially for patients who recently had bariatric surgery

© 2022 Sun JW et al. *JAMA Network Open.*

eTable 2. Study Definitions for Treatment Strategies

Treatment Strategy	Generic Name
Carbamazepine	carbamazepine
Lamotrigine	lamotrigine
Oxcarbazepine	oxcarbazepine
Valproate	divalproex sodium, valproic acid, valproate sodium

To define treatment groups, generic names were mapped to NDC codes.

© 2022 Sun JW et al. *JAMA Network Open.*

eTable 3. List of Baseline and Time-Varying Covariates

Baseline Covariates	
Demographics	Other Comorbidities
Age at initiation	Asthma
Female	Cancer
Year of initiation	Chronic kidney disease*
Comorbidity index (combined score for adults, pediatric comorbidity index for children)[1,2]	Essential tremor*
	Fibromyalgia
Treatment Indications	Sleep disorders
Bipolar disorder	Lifestyle Factors
Epilepsy or convulsions	Alcohol abuse or dependence
Migraine/headache	Drug abuse or dependence
Neuropathic pain	Smoking
Metabolic Conditions	Lab Tests Ordered
Obesity or overweight	Glucose test
Weight management	Hemoglobin A1C test
Abnormal weight gain	Lipid test
Abnormal glucose/Prediabetes	Medications
Metabolic syndrome	Lithium
Hyperinsulinemia	Antipsychotics
Growth conditions	Antidepressants
Hyperlipidemia	Stimulants
Hyperthyroidism	Oral corticosteroids
Hypothyroidism	Weight loss medications*
Nonalcoholic fatty liver disease	Antihypertensives**
Polycystic ovary syndrome	Angiotensin-converting-enzyme inhibitors*
Psychiatric Conditions	Angiotensin II receptor blockers*
Attention-deficit/hyperactivity disorder	Beta blockers*
Anxiety	Thiazides*
Autism and pervasive developmental disorders	Calcium channel blockers*
Delirium	Statins and lipid lowering drugs*
Depression	Healthcare Utilization
Eating disorders	Number of outpatient visits
Psychotic disorders	Number of mental health outpatient visits
Cardiovascular Conditions	Number of distinct generic drugs dispensed
Acute myocardial infarction *	Number of emergency department visits
Coronary artery disease*	Number of hospitalizations
Heart failure*	Any mental health hospitalization
Hemorrhagic stroke*	Days hospitalized
Hypertension	
Ischemic stroke*	
Time-Varying Covariates	
Factors Associated With Weight Change or Type 2 Diabetes	Treatment Indications
Depression	Bipolar disorder
Psychotic disorders	Epilepsy or convulsions
Lithium	Migraine/headache
Antipsychotics	Neuropathic pain
Antidepressants	Healthcare Utilization
Stimulants	Any outpatient visit
Oral corticosteroids	Any emergency department visit
Weight loss medications*	Any hospitalization
Antihypertensives	Any generic drugs dispensed, excluding anticonvulsants
Statins and lipid lowering drugs*	Lifestyle factors
Pregnancy	Alcohol abuse or dependence
Bariatric surgery	Drug abuse or dependence
	Smoking

* Indicates covariate was only included in the adult trial emulation.

© 2022 Sun JW et al. *JAMA Network Open.*

** Indicates covariate was only included in the pediatric trial emulation.

© 2022 Sun JW et al. *JAMA Network Open.*

eMethods. Details on Estimating Inverse Probability Weights and Treatment Effects

We used inverse probability weights to adjust for confounding due to lack of baseline randomization, as well as selection bias due to loss to follow-up [ITT and PP] and treatment nonadherence [PP only].[3–5] The analytic dataset was structured so that each patient had one record for each two-week interval of follow up. In the ITT analysis, follow up ended at the two-week interval of T2D incidence, loss to follow-up, or 5 years, whichever came first. In the PP analysis, we additionally censored patients in the first interval where patients reached the end of the grace period without refilling their initiated medication (treatment discontinuation) or switched treatments (the date a different anticonvulsant medication was dispensed). Therefore, follow up ended at the 2-week interval of T2D incidence, loss to follow up, 5 years, treatment discontinuation, or treatment switch, whichever came first.

Treatment Weights

In both the intention-to-treat and per-protocol analyses, we applied inverse probability of treatment weights (IPTW) to account for baseline confounding.[5] Specifically, we defined the numerator of the weight for a particular individual as the marginal probability of initiating treatment and the denominator of the weight as the probability of initiating that treatment conditional on that individual's levels of the measured baseline covariates. We used multinomial logistic regression to estimate these initiation probabilities. These weights were time-fixed, so each patient had the same IPTW for each 2-week interval of follow up. More details and SAS code for implementation available elsewhere.[5]

Censoring Weights

Intention-to-Treat Analysis

In the intention-to-treat analysis, we applied inverse probability of censoring weights (IPCW) to account for potential selection bias due to loss to follow-up.[5] Probabilities needed for these weights were estimated separately for each treatment group. These weights varied for each individual over time. Therefore, the weight for an individual in a particular 2-week interval of follow-up t, who was still uncensored by t, is a product, over all prior intervals $k<t$, of a ratio of probabilities indexed by interval k: the numerator was an estimate of the marginal probability of remaining uncensored by loss to follow-up through k and the denominator was an estimate of this probability conditional on that individual's level of measured baseline covariates. We used logistic regression to estimate these probabilities, with the censoring indicator as the dependent variable. Patients received a weight of zero once they were censored. Therefore, censored individuals contributed information to the probabilities used for the weight construction up until their censoring time. More details and SAS code for implementation available elsewhere.[5]

Per-Protocol Analysis

In the per-protocol analysis, we applied IPCW to account for potential selection bias due to censoring by treatment discontinuation ($C1_k$), treatment switching ($C2_k$), or loss to follow-up ($C3_k$) by a particular interval k.[5] The same approach above to constructing IPCW for the ITT analysis was used here with a few exceptions.

First, we constructed ICPW separately for each type of censoring (C1, C2, C3). The final IPCW at a particular time was defined as the product of these three weights at that time ($C1_k*C2_k*C3_k$), for individuals still uncensored by that time. As in the ITT analysis, individuals receive a weight of zero at the time of censoring, but they still contribute information to the analysis up until their censoring time. Separate models were used for each censorship reason to allow better prediction of these different types of censorship events.

Second, the denominator of each of the three weights depended not only on baseline covariates (V), but also the history of time-varying covariates to account for potential time-varying confounding. To estimate the probability of remaining uncensored for a given reason in an interval k conditional on baseline and time-varying covariates, we additionally included the values of time-varying covariates corresponding to the current 2-week interval (L_k), the previous 2-week interval (L_{k-1}=lag[L_k]), and an interaction term between these measurements (L_k*lag[L_k]). For time-varying covariates that were chronic conditions, the presence of a diagnosis code was carried forward for the remainder of the follow up period. For example, a patient diagnosed with depression during the 2nd week of follow up was flagged as having depression for each subsequent 2-week interval over follow up. Because we defined treatment discontinuation using the grace period algorithm described in the main text, censoring by treatment discontinuation was only possible during time intervals where a patient's grace period (i.e., allowable gap between prescription fills) would expire if no medication refill were dispensed (G_k=1). Therefore, the weights were not updated during these intervals (as the contribution to the product mentioned above was 1). This approach minimizes model misclassification by incorporating knowledge that this form of censoring was not possible during certain intervals for certain patients. The time k contribution to the denominator of the censoring weights can be summarized as follows:

Treatment discontinuation:

$Pr(C1_k=0|L_k, lag[L_k], L_k*lag[L_k], V, G_k=1)$, if $G_k=1$
1, if $G_k=0$

Treatment switch:

$Pr(C2_k=0|L_k, lag[L_k], L_k*lag[L_k], V)$

Loss to follow up:

$Pr(C3_k=0|L_k, lag[L_k], L_k*lag[L_k], V)$

Third, the numerator of the weights further depended on a subset of the baseline covariates (Z) which were conditioned on in the weighted outcome regression model described below. This provided further weight stabilization at the expense of the assumption that this conditional outcome model was correctly specified.[4] The time k contribution to the numerator of the censoring weights can be summarized as follows:

Treatment discontinuation:

$Pr(C1_k=0|Z, G_k=1)$, if $G_k=1$
1, if $G_k=0$

Treatment switch:

$Pr(C2_k=0|Z)$

Loss to follow up:

$Pr(C3_k=0|Z)$

Estimating Treatment Effects

We estimated the absolute risks of type 2 diabetes for each treatment group using weighted pooled logistic regression models. The weight for each 2-week person-time record in the data was defined as the product of a time-fixed IPTW (i.e., weight remains the same for each person) and time-updated IPCW (i.e., weight changes for each 2-week interval of follow up). The weights were truncated at the 1st and 99th percentiles to prevent outliers from influencing the analysis.[6] The dependent variable in this pooled over interval model was T2D and the independent variables were indicators for which treatment the patient initiated and a function of time (modelled flexibly as linear and quadratic terms, as well as interactions terms with treatment to minimize misclassification of the baseline hazard). In the PP analysis, the independent variables also included subset of the baseline covariates Z that were included in the numerator of the censoring weights described above.

From these models, we predicted the hazard of T2D under each treatment strategy for each 2-week interval of follow up, which can be used to estimate the cumulative survival under initiation of or adherence to each treatment strategy.[7] We generated adjusted survival curves for the probability of remaining free of type 2 diabetes over follow up. In the per-protocol analysis, these survival curves were standardized by the subset of baseline covariates Z that were used to estimate the numerator of the censoring weights.[8] We estimated 95% confidence intervals for the 2-year and 5-year risk (complement of survival) differences using nonparametric bootstrapping of 400 samples. The desired interpretation of our estimates relied on the assumptions that our weight models were correctly specified, the hazard models were a correctly specified function of time (and Z), and that measured baseline and time-varying covariates were sufficient to control confounding due to lack of baseline randomization and selection bias due to loss to follow-up. Additional details on these analyses and how to implement them are available elsewhere.[8]

© 2022 Sun JW et al. *JAMA Network Open.*

eFigure 1. Flow Diagram of Cohort Assembly

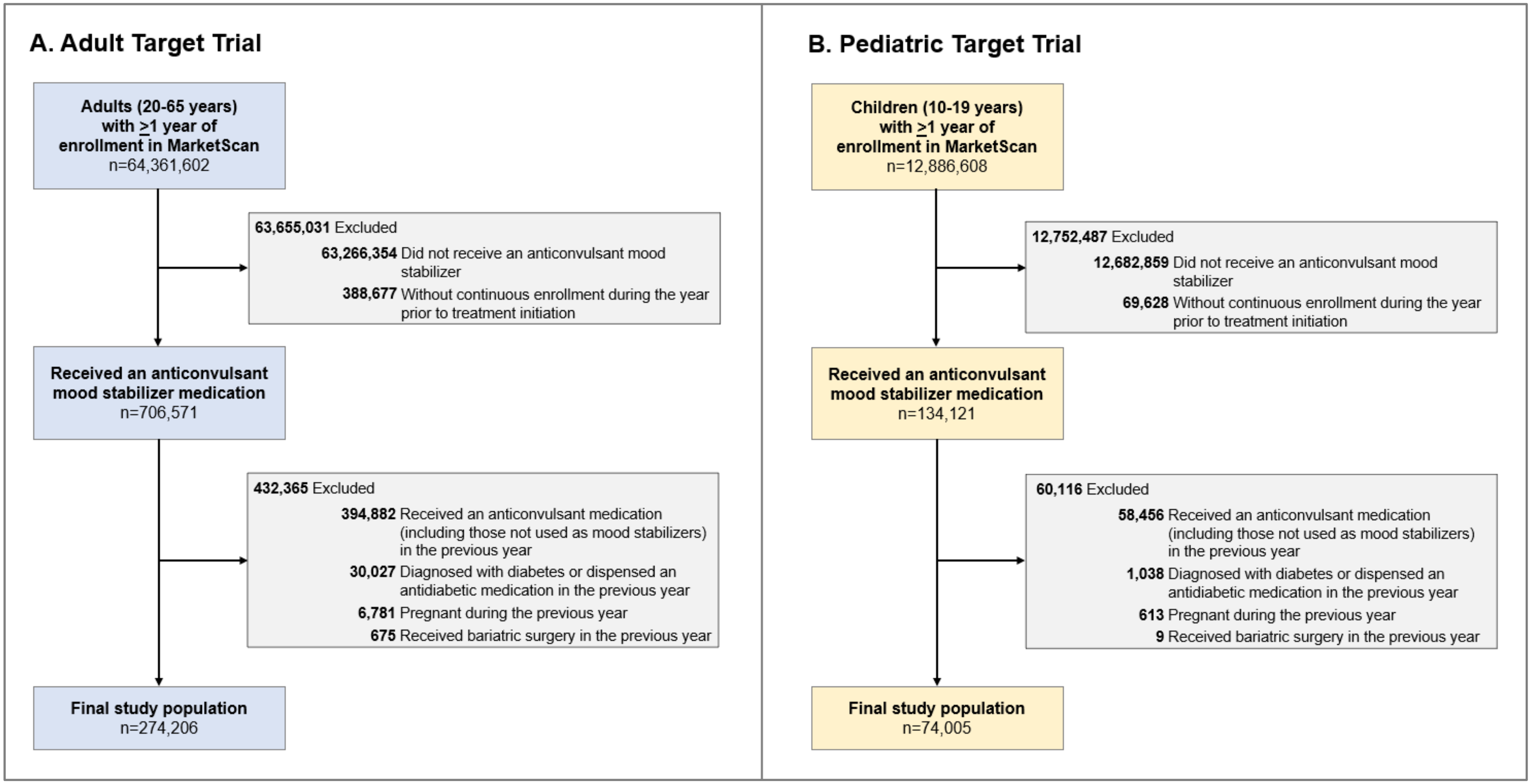

© 2022 Sun JW et al. *JAMA Network Open.*

eTable 4. Unadjusted Baseline Characteristics of Adults Who Initiated Anticonvulsant Mood Stabilizer Treatment in MarketScan

Characteristic	Carbamazepine		Lamotrigine		Oxcarbazepine		Valproate	
	N	%	N	%	N	%	N	%
No. of patients	26641	100.0	132739	100.0	24226	100.0	90600	100.0
Demographics								
Age at initiation, years								
mean (SD)	45.45	12.5	38.02	12.8	39.49	13.4	41.04	13.3
20-39 years	8339	31.3	73698	55.5	12204	50.4	41544	45.9
40-54 years	10566	39.7	41448	31.2	7835	32.3	30810	34.0
55-65 years	7736	29	17593	13.3	4187	17.3	18246	20.1
Female	16069	60.3	87039	65.6	14418	59.5	41902	46.2
Year of initiation								
2011	4839	18.2	19910	15.0	3525	14.6	25739	28.4
2012	5141	19.3	22776	17.2	4194	17.3	17027	18.8
2013	3878	14.6	17784	13.4	3222	13.3	11272	12.4
2014	3314	12.4	16195	12.2	3037	12.5	9560	10.6
2015	2663	10.0	13228	10.0	2438	10.1	7555	8.3
2016	2386	9.0	13589	10.2	2512	10.4	7085	7.8
2017	2026	7.6	12753	9.6	2378	9.8	5521	6.1
2018	1907	7.2	12773	9.6	2259	9.3	5342	5.9
2019	487	1.8	3731	2.8	661	2.7	1499	1.7
Combined comorbidity index; mean (SD)	0.51	1.2	0.95	1.0	0.92	1.2	0.88	1.3
Treatment Indications								
Bipolar disorder	3669	13.8	51393	38.7	8445	34.9	32447	35.8
Epilepsy or convulsions	2156	8.1	5715	4.3	1680	6.9	9458	10.4
Migraine/headache	6917	26.0	19657	14.8	4298	17.7	25769	28.4
Neuropathic pain	10876	40.8	13913	10.5	4913	20.3	10156	11.2
Metabolic Conditions								
Obesity or overweight	2931	11.0	15097	11.4	2971	12.3	8258	9.1
Weight management	313	1.2	2142	1.6	335	1.4	861	1.0
Abnormal weight gain	446	1.7	3337	2.5	466	1.9	1356	1.5
Abnormal glucose/Prediabetes	1251	4.7	5096	3.8	1031	4.3	3556	3.9
Metabolic syndrome	154	0.6	801	0.6	144	0.6	391	0.4
Hyperinsulinemia	13	0.0	130	0.1	21	0.1	84	0.1
Growth conditions	213	0.8	1008	0.8	200	0.8	759	0.8
Hyperlipidemia	6325	23.7	22000	16.6	4603	19.0	19469	21.5
Hyperthyroidism	161	0.6	844	0.6	157	0.6	628	0.7
Hypothyroidism	2725	10.2	12644	9.5	2350	9.7	8518	9.4
Nonalcoholic fatty liver disease	284	1.1	1018	0.8	229	0.9	813	0.9
Polycystic ovary syndrome	144	0.5	1532	1.2	218	0.9	344	0.4
Lab Tests Ordered								
Glucose test	1854	7.0	8243	6.2	1574	6.5	6474	7.1
Hemoglobin A1C test	3496	13.1	17938	13.5	3323	13.7	10567	11.7
Lipid test	11052	41.5	50344	37.9	9359	38.6	35261	38.9
Psychiatric Conditions								
ADHD	840	3.2	13104	9.9	2032	8.4	4610	5.1
Anxiety	5868	22.0	61497	46.3	9560	39.5	27069	29.9
Autism and pervasive developmental disorders	105	0.4	641	0.5	245	1.0	953	1.1
Delirium	223	0.8	753	0.6	225	0.9	1473	1.6
Depression	6416	24.1	77693	58.5	11144	46.0	33280	36.7
Eating disorders	96	0.4	2142	1.6	223	0.9	405	0.4
Psychotic disorders	915	3.4	5403	4.1	1745	7.2	9770	10.8
Cardiovascular Conditions								

© 2022 Sun JW et al. *JAMA Network Open.*

Acute myocardial infarction	95	0.4	253	0.2	62	0.3	350	0.4
Coronary artery disease	858	3.2	2412	1.8	618	2.6	2932	3.2
Heart failure	285	1.1	883	0.7	246	1.0	1223	1.3
Hemorrhagic stroke	138	0.5	343	0.3	105	0.4	1025	1.1
Hypertension	7080	26.6	22407	16.9	5316	21.9	21379	23.6
Ischemic stroke	391	1.5	1045	0.8	376	1.6	1708	1.9
Other Comorbidities								
Asthma	1899	7.1	10317	7.8	1977	8.2	6718	7.4
Cancer	967	3.6	2706	2.0	649	2.7	2460	2.7
Chronic kidney disease	321	1.2	1090	0.8	270	1.1	1449	1.6
Essential tremor	136	0.5	524	0.4	107	0.4	663	0.7
Fibromyalgia	1562	5.9	6385	4.8	1277	5.3	4280	4.7
Sleep disorders	3730	14.0	24994	18.8	4316	17.8	15668	17.3
Lifestyle Factors								
Alcohol abuse or dependence	1791	6.7	8892	6.7	2315	9.6	7437	8.2
Drug abuse or dependence	2006	7.5	10279	7.7	3072	12.7	10298	11.4
Smoking	3145	11.8	14376	10.8	3558	14.7	12826	14.2
Medications								
Lithium	630	2.4	6706	5.1	1047	4.3	4048	4.5
Antipsychotics	3121	11.7	35639	26.8	6671	27.5	26468	29.2
Antidepressants	8318	31.2	80521	60.7	12359	51.0	39202	43.3
Stimulants	2152	8.1	24058	18.1	3886	16.0	10111	11.2
Oral corticosteroids	7161	26.9	24830	18.7	5465	22.6	18346	20.2
Weight loss medications	143	0.5	925	0.7	131	0.5	403	0.4
ACE inhibitors	2796	10.5	8738	6.6	1994	8.2	8312	9.2
ARBs	1673	6.3	5029	3.8	1235	5.1	4202	4.6
Beta blockers	3342	12.5	13304	10.0	2822	11.6	12861	14.2
Thiazides	2899	10.9	8796	6.6	1985	8.2	7496	8.3
Calcium channel blockers	1831	6.9	5176	3.9	1342	5.5	6358	7.0
Statins and lipid lowering drugs	4375	16.4	13612	10.3	2908	12.0	13650	15.1
Healthcare Utilization								
Number of outpatient visits; median (IQR)	9	(5-18)	12	(6-23)	11	(6-21)	10	(5-19)
Number of mental health outpatient visits; median (IQR)	0	(0-2)	4	(1-10)	3	(0-8)	2	(0-6)
Number of distinct generic drugs dispensed; median (IQR)	7	(3-11)	7	(4-11)	7	(4-12)	7	(4-12)
Number of ED visits								
0	17070	64.1	90620	68.3	14897	61.5	55947	61.8
1	5528	20.7	24515	18.5	5050	20.8	18138	20.0
≥2	4043	15.2	17604	13.3	4279	17.7	16515	18.2
Number of hospitalizations								
0	22542	84.6	115347	86.9	18924	78.1	69801	77.0
1	2892	10.9	13381	10.1	3858	15.9	14608	16.1
≥2	1207	4.5	4011	3.0	1444	6.0	6191	6.8
Any mental health hospitalization	2456	9.2	11835	8.9	3834	15.8	13949	15.4
Days hospitalized								
0	22542	84.6	115347	86.9	18924	78.1	69801	77.0
1 to 5	1725	6.5	8303	6.3	1932	8.0	7219	8.0
≥6	2374	8.9	9089	6.8	3370	13.9	13580	15.0

Abbreviations: ACE=Angiotensin-converting-enzyme; ARBs=Angiotensin II receptor blockers; ADHD=Attention-deficit/hyperactivity disorder; ED=Emergency department; IQR=Interquartile range; SD=Standard deviation

© 2022 Sun JW et al. *JAMA Network Open.*

eTable 5. Unadjusted Baseline Characteristics of Children Who Initiated Anticonvulsant Mood Stabilizer Treatment in MarketScan

Characteristic	Carbamazepine		Lamotrigine		Oxcarbazepine		Valproate	
	N	%	N	%	N	%	N	%
No. of patients	2532	100.0	36394	100.0	12434	100.0	22645	100.0
Demographics								
Age at initiation (years)								
mean (SD)	15.63	2.7	16.01	2.4	14.69	2.7	15.51	2.7
10-12 years	386	15.2	3564	9.8	3100	24.9	3761	16.6
13-17 years	1369	54.1	20949	57.6	7070	56.9	12382	54.7
18-19 years	777	30.7	11881	32.6	2264	18.2	6502	28.7
Female	1193	47.1	24273	66.7	6023	48.4	7183	31.7
Year of initiation								
2011	547	21.6	4902	13.5	1854	14.9	6110	27.0
2012	531	21.0	5642	15.5	2056	16.5	4329	19.1
2013	366	14.5	4767	13.1	1572	12.6	2853	12.6
2014	318	12.6	4468	12.3	1619	13.0	2454	10.8
2015	231	9.1	3781	10.4	1277	10.3	1837	8.1
2016	218	8.6	3877	10.7	1335	10.7	1761	7.8
2017	151	6.0	3882	10.7	1233	9.9	1520	6.7
2018	135	5.3	3921	10.8	1140	9.2	1403	6.2
2019	35	1.4	1154	3.2	348	2.8	378	1.7
Pediatric comorbidity index; mean (SD)	5.97	4.3	6.51	3.8	6.06	3.9	5.83	4.1
Treatment Indications								
Bipolar disorder	968	38.2	14723	40.5	4556	36.6	8840	39.0
Epilepsy or convulsions	344	13.6	2204	6.1	1862	15.0	3627	16.0
Migraine/headache	415	16.4	4679	12.9	1403	11.3	4835	21.4
Neuropathic pain	176	7.0	1155	3.2	279	2.2	569	2.5
Metabolic Conditions								
Obesity or overweight	161	6.4	2649	7.3	862	6.9	1117	4.9
Weight management	62	2.4	1488	4.1	498	4.0	669	3.0
Abnormal weight gain	45	1.8	689	1.9	235	1.9	253	1.1
Abnormal glucose/Prediabetes	22	0.9	315	0.9	92	0.7	175	0.8
Metabolic syndrome	7	0.3	134	0.4	40	0.3	61	0.3
Growth conditions	23	0.9	210	0.6	100	0.8	175	0.8
Hyperlipidemia	54	2.1	734	2.0	226	1.8	408	1.8
Hyperthyroidism or Hypothyroidism	58	2.3	1010	2.8	249	2.0	543	2.4
Lab Tests Ordered								
Glucose test	164	6.5	2081	5.7	795	6.4	1418	6.3
Hemoglobin A1C test	156	6.2	2798	7.7	830	6.7	1382	6.1
Lipid test	453	17.9	7248	19.9	2198	17.7	4108	18.1
Psychiatric Conditions								
ADHD	439	17.3	7155	19.7	2880	23.2	4024	17.8
Anxiety	714	28.2	17075	46.9	4235	34.1	5976	26.4
Autism and pervasive developmental disorders	209	8.3	1878	5.2	1262	10.1	2458	10.9
Delirium	38	1.5	375	1.0	159	1.3	331	1.5
Depression	975	38.5	22100	60.7	5477	44.0	8123	35.9
Eating disorders	44	1.7	1537	4.2	195	1.6	208	0.9
Psychotic disorders	257	10.2	2694	7.4	1092	8.8	2755	12.2
Other Comorbidities								
Asthma	309	12.2	3813	10.5	1428	11.5	2510	11.1
Cancer	19	0.8	102	0.3	65	0.5	103	0.5
Fibromyalgia	62	2.4	641	1.8	167	1.3	313	1.4

© 2022 Sun JW et al. *JAMA Network Open.*

Hypertension	42	1.7	459	1.3	181	1.5	451	2.0
Sleep disorders	221	8.7	3647	10.0	1042	8.4	1886	8.3
Lifestyle Factors								
Alcohol abuse or dependence	147	5.8	1463	4.0	479	3.9	1060	4.7
Drug abuse or dependence	364	14.4	3675	10.1	1306	10.5	3023	13.3
Smoking	189	7.5	1709	4.7	626	5.0	1457	6.4
Medications								
Lithium	117	4.6	1519	4.2	332	2.7	847	3.7
Antipsychotics	913	36.1	12898	35.4	4527	36.4	9089	40.1
Antidepressants	1001	39.5	21777	59.8	5760	46.3	8272	36.5
Stimulants	807	31.9	11306	31.1	4793	38.5	8010	35.4
Oral corticosteroids	329	13.0	4210	11.6	1319	10.6	2706	11.9
Antihypertensives	468	18.5	5679	15.6	2750	22.1	4681	20.7
Healthcare Utilization								
Number of outpatient visits; median (IQR)	9	(5-19)	13	(7-26)	11	(5-21)	9	(5-18)
Number of mental health outpatient visits; median (IQR)	3	(0-9)	6	(2-17)	4	(1-12)	3	(0-10)
Number of distinct generic drugs dispensed; median (IQR)	5	(2-8)	5	(3-8)	4	(2-7)	5	(3-8)
Number of ED visits								
0	1299	51.3	21279	58.5	6810	54.8	12229	54.0
1	634	25.0	8455	23.2	3067	24.7	5396	23.8
≥2	599	23.7	6660	18.3	2557	20.6	5020	22.2
Number of hospitalizations								
0	1733	68.4	27510	75.6	8752	70.4	15993	70.6
1	554	21.9	6368	17.5	2600	20.9	4573	20.2
≥2	245	9.7	2516	6.9	1082	8.7	2079	9.2
Any mental health hospitalization	681	26.9	8074	22.2	3096	24.9	5528	24.4
Days hospitalized								
0	1733	68.4	27510	75.6	8752	70.4	15993	70.6
1 to 5	209	8.3	2588	7.1	1084	8.7	1704	7.5
≥6	590	23.3	6296	17.3	2598	20.9	4948	21.9

Abbreviations: ADHD=Attention-deficit/hyperactivity disorder; ED=Emergency department; IQR=Interquartile range; SD=Standard deviation

© 2022 Sun JW et al. *JAMA Network Open.*

eTable 6. Baseline Characteristics of Adults Who Initiated Anticonvulsant Mood Stabilizer Treatment in MarketScan After Accounting for Baseline Confounding

Characteristic	Carbamazepine		Lamotrigine		Oxcarbazepine		Valproate	
	N	%	N	%	N	%	N	%
No. of patients	26806	100.0	130670	100.0	24712	100.0	89831	100.0
Demographics								
Age at initiation, years								
mean (SD)	39.52	13.1	39.67	13.0	39.77	13.5	39.78	13.1
20-39 years	13507	50.4	65468	50.1	12298	49.8	44877	50.0
40-54 years	8892	33.2	43373	33.2	8014	32.4	29336	32.7
55-65 years	4408	16.4	21829	16.7	4401	17.8	15619	17.4
Female	15123	56.4	75729	58.0	14417	58.3	51569	57.4
Year of initiation								
2011	5516	20.6	25525	19.5	4848	19.6	17827	19.8
2012	5143	19.2	23618	18.1	4475	18.1	16296	18.1
2013	3667	13.7	17224	13.2	3290	13.3	11978	13.3
2014	3306	12.3	15365	11.8	2897	11.7	10568	11.8
2015	2509	9.4	12334	9.4	2314	9.4	8481	9.4
2016	2387	8.9	12150	9.3	2298	9.3	8249	9.2
2017	1993	7.4	10862	8.3	2027	8.2	7268	8.1
2018	1790	6.7	10556	8.1	1985	8.0	7098	7.9
2019	495	1.8	3036	2.3	578	2.3	2066	2.3
Combined comorbidity index; mean (SD)	1.01	1.3	0.91	1.1	0.9	1.2	0.91	1.2
Treatment Indications								
Bipolar disorder	10829	40.4	48095	36.8	8973	36.3	33303	37.1
Epilepsy or convulsions	2329	8.7	9649	7.4	1763	7.1	6393	7.1
Migraine/headache	6083	22.7	26740	20.5	5205	21.1	18972	21.1
Neuropathic pain	3596	13.4	18084	13.8	3819	15.5	12792	14.2
Metabolic Conditions								
Obesity or overweight	2923	10.9	14290	10.9	2660	10.8	9816	10.9
Weight management	341	1.3	1767	1.4	325	1.3	1176	1.3
Abnormal weight gain	570	2.1	2740	2.1	516	2.1	1850	2.1
Abnormal glucose/Prediabetes	1209	4.5	5284	4.0	993	4.0	3648	4.1
Metabolic syndrome	167	0.6	723	0.6	139	0.6	484	0.5
Hyperinsulinemia	34	0.1	129	0.1	23	0.1	79	0.1
Growth conditions	258	1.0	1091	0.8	200	0.8	721	0.8
Hyperlipidemia	5324	19.9	24995	19.1	4752	19.2	17327	19.3
Hyperthyroidism	198	0.7	875	0.7	166	0.7	614	0.7
Hypothyroidism	2613	9.7	12573	9.6	2371	9.6	8558	9.5
Nonalcoholic fatty liver disease	289	1.1	1173	0.9	214	0.9	797	0.9
Polycystic ovary syndrome	231	0.9	1087	0.8	211	0.9	695	0.8
Lab Tests Ordered								
Glucose test	1887	7.0	8767	6.7	1668	6.7	6047	6.7
Hemoglobin A1C test	3580	13.4	17202	13.2	3222	13.0	11730	13.1
Lipid test	10296	38.4	50624	38.7	9589	38.8	34818	38.8
Psychiatric Conditions								
ADHD	2046	7.6	10181	7.8	1917	7.8	6928	7.7
Anxiety	10898	40.7	51181	39.2	9602	38.9	35189	39.2
Autism and pervasive developmental disorders	216	0.8	965	0.7	181	0.7	663	0.7
Delirium	420	1.6	1314	1.0	255	1.0	933	1.0
Depression	13280	49.5	63468	48.6	11824	47.8	43411	48.3
Eating disorders	263	1.0	1411	1.1	282	1.1	863	1.0
Psychotic disorders	2274	8.5	8904	6.8	1712	6.9	6077	6.8
Cardiovascular Conditions								

© 2022 Sun JW et al. *JAMA Network Open.*

Acute myocardial infarction	85	0.3	372	0.3	69	0.3	259	0.3
Coronary artery disease	706	2.6	3284	2.5	612	2.5	2317	2.6
Heart failure	305	1.1	1285	1.0	234	0.9	900	1.0
Hemorrhagic stroke	244	0.9	806	0.6	155	0.6	553	0.6
Hypertension	6052	22.6	27070	20.7	5096	20.6	18808	20.9
Ischemic stroke	413	1.5	1793	1.4	334	1.3	1210	1.3
Other Comorbidities								
Asthma	2267	8.5	10221	7.8	1909	7.7	7081	7.9
Cancer	724	2.7	3190	2.4	613	2.5	2230	2.5
Chronic kidney disease	382	1.4	1559	1.2	284	1.2	1064	1.2
Essential tremor	164	0.6	701	0.5	132	0.5	476	0.5
Fibromyalgia	1625	6.1	6726	5.1	1233	5.0	4645	5.2
Sleep disorders	5380	20.1	24006	18.4	4468	18.1	16650	18.5
Lifestyle Factors								
Alcohol abuse or dependence	2699	10.1	10364	7.9	1875	7.6	7302	8.1
Drug abuse or dependence	3478	13.0	13049	10.0	2368	9.6	9080	10.1
Smoking	4022	15.0	16851	12.9	3128	12.7	11729	13.1
Medications								
Lithium	1689	6.3	6605	5.1	1206	4.9	4614	5.1
Antipsychotics	8547	31.9	35833	27.4	6727	27.2	24655	27.4
Antidepressants	14684	54.8	68947	52.8	12817	51.9	47217	52.6
Stimulants	4453	16.6	19876	15.2	3715	15.0	13722	15.3
Oral corticosteroids	5664	21.1	26295	20.1	5047	20.4	18370	20.4
Weight loss medications	147	0.5	781	0.6	146	0.6	542	0.6
ACE inhibitors	2256	8.4	10469	8.0	1986	8.0	7270	8.1
ARBs	1202	4.5	5777	4.4	1095	4.4	4092	4.6
Beta blockers	3526	13.2	15449	11.8	2917	11.8	10627	11.8
Thiazides	2175	8.1	10060	7.7	1922	7.8	7037	7.8
Calcium channel blockers	1571	5.9	6947	5.3	1314	5.3	4850	5.4
Statins and lipid lowering drugs	3468	12.9	16398	12.5	3141	12.7	11388	12.7
Healthcare Utilization								
Number of outpatient visits; median (IQR)	12	(6-23)	12	(6-22)	11	(6-21)	11	(5-21)
Number of mental health outpatient visits; median (IQR)	3	(0-9)	3	(1-9)	3	(0-8)	3	(0-8)
Number of distinct generic drugs dispensed; median (IQR)	8	(4-12)	7	(4-12)	7	(4-11)	7	(4-11)
Number of ED visits								
0	16247	60.6	83639	64.0	15946	64.5	57133	63.6
1	5422	20.2	25778	19.7	4798	19.4	17936	20.0
≥2	5139	19.2	21253	16.3	3969	16.1	14763	16.4
Number of hospitalizations								
0	20629	77.0	106890	81.8	20356	82.4	73396	81.7
1	4268	15.9	17261	13.2	3161	12.8	11899	13.2
≥2	1910	7.1	6519	5.0	1196	4.8	4537	5.1
Any mental health hospitalization	4299	16.0	16171	12.4	2934	11.9	11137	12.4
Days hospitalized								
0	20629	77.0	106890	81.8	20356	82.4	73396	81.7
1 to 5	2321	8.7	9576	7.3	1744	7.1	6663	7.4
≥6	3857	14.4	14204	10.9	2612	10.6	9772	10.9

Abbreviations: ACE=Angiotensin-converting-enzyme; ARBs=Angiotensin II receptor blockers; ADHD=Attention-deficit/hyperactivity disorder; ED=Emergency department; IQR=Interquartile range; SD=Standard deviation

Estimates weighted by the inverse probability of treatment.

© 2022 Sun JW et al. *JAMA Network Open.*

eTable 7. Baseline Characteristics of Children Who Initiated Anticonvulsant Mood Stabilizer Treatment in MarketScan After Accounting for Baseline Confounding

Characteristic	Carbamazepine		Lamotrigine		Oxcarbazepine		Valproate	
	N	%	N	%	N	%	N	%
No. of patients	2548	100.0	36136	100.0	12495	100.0	21922	100.0
Demographics								
Age at initiation (years)								
mean (SD)	15.67	2.7	15.62	2.6	15.64	2.6	15.59	2.6
10-12 years	372	14.6	5297	14.7	1818	14.5	3352	15.3
13-17 years	1373	53.9	20374	56.4	7109	56.9	12145	55.4
18-19 years	804	31.5	10467	29	3568	28.6	6432	29.3
Female	1320	51.8	18975	52.5	6627	53.0	11058	50.4
Year of initiation								
2011	466	18.3	6484	17.9	2290	18.3	4107	18.7
2012	439	17.2	6233	17.2	2124	17.0	3929	17.9
2013	336	13.2	4713	13.0	1620	13.0	2892	13.2
2014	303	11.9	4349	12.0	1497	12.0	2616	11.9
2015	253	9.9	3429	9.5	1176	9.4	2096	9.6
2016	247	9.7	3543	9.8	1255	10.0	2082	9.5
2017	230	9.0	3272	9.1	1123	9.0	1890	8.6
2018	214	8.4	3200	8.9	1084	8.7	1821	8.3
2019	60	2.4	913	2.5	326	2.6	489	2.2
Pediatric comorbidity index; mean (SD)	6.41	4.2	6.25	4.0	6.34	4.0	6.29	4.0
Treatment Indications								
Bipolar disorder	1073	42.1	14461	40.0	5110	40.9	8960	40.9
Epilepsy or convulsions	277	10.9	3838	10.6	1310	10.5	2514	11.5
Migraine/headache	378	14.8	5535	15.3	1904	15.2	3539	16.1
Neuropathic pain	79	3.1	1070	3.0	388	3.1	644	2.9
Metabolic Conditions								
Obesity or overweight	160	6.3	2377	6.6	834	6.7	1387	6.3
Weight management	85	3.3	1318	3.6	454	3.6	745	3.4
Abnormal weight gain	39	1.5	624	1.7	200	1.6	375	1.7
Abnormal glucose/Prediabetes	22	0.9	300	0.8	95	0.8	188	0.9
Metabolic syndrome	7	0.3	123	0.3	41	0.3	74	0.3
Growth conditions	16	0.6	257	0.7	88	0.7	171	0.8
Hyperlipidemia	47	1.8	704	1.9	245	2.0	421	1.9
Hyperthyroidism or Hypothyroidism	73	2.9	908	2.5	313	2.5	530	2.4
Lab Tests Ordered								
Glucose test	145	5.7	2205	6.1	768	6.1	1366	6.2
Hemoglobin A1C test	176	6.9	2554	7.1	880	7.0	1531	7.0
Lipid test	464	18.2	6902	19.1	2400	19.2	4176	19.0
Psychiatric Conditions								
ADHD	503	19.7	7175	19.9	2509	20.1	4328	19.7
Anxiety	969	38.0	13810	38.2	4846	38.8	8048	36.7
Autism and pervasive developmental disorders	201	7.9	3003	8.3	1015	8.1	1844	8.4
Delirium	35	1.4	445	1.2	153	1.2	277	1.3
Depression	1289	50.6	18137	50.2	6352	50.8	10715	48.9
Eating disorders	78	3.1	978	2.7	346	2.8	475	2.2
Psychotic disorders	241	9.4	3347	9.3	1169	9.4	2078	9.5
Other Comorbidities								
Asthma	282	11.1	3939	10.9	1364	10.9	2391	10.9
Cancer	10	0.4	125	0.3	47	0.4	93	0.4
Fibromyalgia	43	1.7	588	1.6	203	1.6	345	1.6

© 2022 Sun JW et al. *JAMA Network Open.*

Hypertension	44	1.7	534	1.5	192	1.5	338	1.5
Sleep disorders	239	9.4	3428	9.5	1172	9.4	2112	9.6
Lifestyle Factors								
Alcohol abuse or dependence	114	4.5	1566	4.3	576	4.6	973	4.4
Drug abuse or dependence	311	12.2	4163	11.5	1489	11.9	2569	11.7
Smoking	139	5.4	1979	5.5	708	5.7	1228	5.6
Medications								
Lithium	114	4.5	1460	4.0	493	3.9	954	4.4
Antipsychotics	998	39.1	13727	38.0	4818	38.6	8435	38.5
Antidepressants	1297	50.9	18251	50.5	6360	50.9	10819	49.4
Stimulants	878	34.4	12355	34.2	4306	34.5	7551	34.4
Oral corticosteroids	304	11.9	4170	11.5	1463	11.7	2545	11.6
Antihypertensives	471	18.5	6764	18.7	2344	18.8	4079	18.6
Healthcare Utilization								
Number of outpatient visits; median (IQR)	11	(6-23)	12	(6-23)	12	(6-23)	11	(5-22)
Number of mental health outpatient visits; median (IQR)	5	(1-14)	5	(1-14)	5	(1-14)	4	(1-12)
Number of distinct generic drugs dispensed; median (IQR)	5	(3-8)	5	(3-8)	5	(3-8)	5	(3-8)
Number of ED visits								
0	1412	55.4	20211	55.9	6973	55.8	12020	54.8
1	621	24.4	8643	23.9	2968	23.8	5289	24.1
≥2	516	20.3	7281	20.1	2554	20.4	4613	21.0
Number of hospitalizations								
0	1845	72.4	26484	73.3	9040	72.3	15899	72.5
1	495	19.4	6801	18.8	2412	19.3	4199	19.2
≥2	208	8.2	2850	7.9	1043	8.4	1824	8.3
Any mental health hospitalization	614	24.1	8457	23.4	3017	24.1	5191	23.7
Days hospitalized								
0	1845	72.4	26484	73.3	9040	72.3	15899	72.5
1 to 5	196	7.7	2716	7.5	948	7.6	1705	7.8
≥6	507	19.9	6935	19.2	2508	20.1	4318	19.7

Abbreviations: ADHD=Attention-deficit/hyperactivity disorder; ED=Emergency department; IQR=Interquartile range; SD=Standard deviation
Estimates weighted by the inverse probability of treatment.

© 2022 Sun JW et al. *JAMA Network Open.*

eTable 8. Absolute Rate of Type 2 Diabetes Among Patients Within the Adult and Pediatric Trial Emulations

Treatment Group	No. of patients	No. of events	Total follow up in person-years	Mean follow up in person-years (SD)	Incidence rate per 1,000 person-years
**Adult Trial**					
Intention-to-treat					
Carbamazepine	26,641	932	52,438	2.0 (1.6)	17.77
Lamotrigine	132,739	3,377	237,867	1.8 (1.5)	14.20
Oxcarbazepine	24,226	752	43,874	1.8 (1.5)	17.14
Valproate	90,600	3,371	175,972	1.9 (1.6)	19.16
Per-protocol					
Carbamazepine	26,641	220	9,193	0.35 (0.59)	23.93
Lamotrigine	132,739	1,160	79,091	0.60 (0.79)	14.67
Oxcarbazepine	24,226	202	9,998	0.41 (0.60)	20.20
Valproate	90,600	803	36,988	0.41 (0.66)	21.71
**Pediatric Trial**					
Intention-to-treat					
Carbamazepine	2,532	14	5,547	2.2 (1.7)	2.52
Lamotrigine	36,394	160	75,561	2.1 (1.6)	2.12
Oxcarbazepine	12,434	54	26,073	2.1 (1.6)	2.07
Valproate	22,645	105	50,455	2.2 (1.7)	2.08
Per-protocol					
Carbamazepine	2,532	6	1,084	0.43 (0.63)	5.54
Lamotrigine	36,394	57	22,463	0.62 (0.79)	2.54
Oxcarbazepine	12,434	17	6,812	0.55 (0.75)	2.50
Valproate	22,645	30	7,990	0.35 (0.58)	3.75

© 2022 Sun JW et al. *JAMA Network Open.*

eTable 9. Hazard Ratios and 95% Confidence Intervals Comparing the Incidence of Type 2 Diabetes Across Mood Stabilizer Treatment, By Level of Adjustment

Level of Adjustment	Carbamazepine	Lamotrigine	Oxcarbazepine	Valproate
**Adult Trial**				
Intention-to-treat				
Crude	1.26 (1.17-1.35)	ref	1.21 (1.12-1.31)	1.36 (1.29-1.42)
Fully adjusted for baseline covariates	1.07 (0.96-1.19)	ref	1.07 (0.98-1.17)	1.15 (1.09-1.22)
Per-protocol				
Crude	1.55 (1.34-1.79)	ref	1.31 (1.13-1.53)	1.44 (1.31-1.57)
Partially adjusted for baseline covariates	1.25 (0.99-1.59)	ref	1.05 (0.89-1.24)	1.20 (1.06-1.36)
Fully adjusted for baseline and time-varying covariates	1.17 (0.93-1.46)	ref	1.08 (0.91-1.29)	1.14 (1.01-1.28)
**Pediatric Trial**				
Intention-to-treat				
Crude	1.21 (0.70-2.09)	ref	0.98 (0.72-1.34)	1.00 (0.78-1.27)
Fully adjusted for baseline covariates	1.04 (0.58-1.84)	ref	1.11 (0.78-1.56)	1.18 (0.87-1.60)
Per-protocol				
Crude	1.87 (0.81-4.34)	ref	0.94 (0.54-1.61)	1.23 (0.79-1.92)
Partially adjusted for baseline covariates	1.38 (0.55-3.46)	ref	1.18 (0.56-2.50)	1.46 (0.77-2.76)
Fully adjusted for baseline and time-varying covariates	1.23 (0.48-3.14)	ref	0.84 (0.44-1.61)	1.39 (0.75-2.56)

Estimates were weighted by the inverse probability of treatment and the inverse probability or censoring. We truncated weights at the 1st and 99th percentiles.

© 2022 Sun JW et al. *JAMA Network Open.*

eTable 10. Adjusted Hazard Ratios and 95% Confidence Intervals Comparing the Incidence of Type 2 Diabetes Across Mood Stabilizer Treatment in Adults, Stratified by Age and Treatment Indication

Subgroup	Carbamazepine	Lamotrigine	Oxcarbazepine	Valproate
Intention-to-treat				
Total population	1.07 (0.96-1.20)	ref	1.07 (0.98-1.17)	1.16 (1.09-1.22)
Age 20-39 years	1.21 (0.95-1.52)	ref	1.09 (0.92-1.30)	1.25 (1.12-1.39)
Age 40-54 years	1.10 (0.94-1.28)	ref	1.06 (0.93-1.21)	1.13 (1.04-1.23)
Age 55-65 years	0.93 (0.77-1.12)	ref	1.07 (0.90-1.27)	1.12 (1.01-1.25)
Bipolar disorder	1.15 (0.94-1.41)	ref	1.10 (0.95-1.27)	1.24 (1.14-1.36)
Epilepsy	1.06 (0.72-1.57)	ref	1.08 (0.72-1.63)	1.33 (1.04-1.70)
Per-protocol				
Total population	1.17 (0.93-1.46)	ref	1.08 (0.91-1.29)	1.14 (1.01-1.28)
Age 20-39 years	1.54 (0.97-2.44)	ref	0.98 (0.66-1.44)	1.21 (0.95-1.55)
Age 40-54 years	1.27 (0.91-1.79)	ref	1.06 (0.82-1.36)	1.23 (1.04-1.46)
Age 55-65 years	0.82 (0.57-1.17)	ref	1.20 (0.90-1.60)	1.04 (0.95-1.26)
Bipolar disorder	1.44 (0.96-2.17)	ref	1.08 (0.81-1.44)	1.16 (0.97-1.38)
Epilepsy	0.94 (0.48-1.86)	ref	0.75 (0.35-1.59)	1.54 (0.96-2.46)

Adjusted estimates were weighted by the inverse probability of treatment and the inverse probability or censoring. Subgroup analyses were not conducted in the pediatric trial emulation due to lack of power.

© 2022 Sun JW et al. *JAMA Network Open.*

eFigure 2. Sensitivity Analysis Evaluating the Potential Role of Unmeasured Confounding on Observed Point Estimates in the Intention-to-Treat Analysis

A. Adult Trial Emulation *B. Pediatric Trial Emulation*

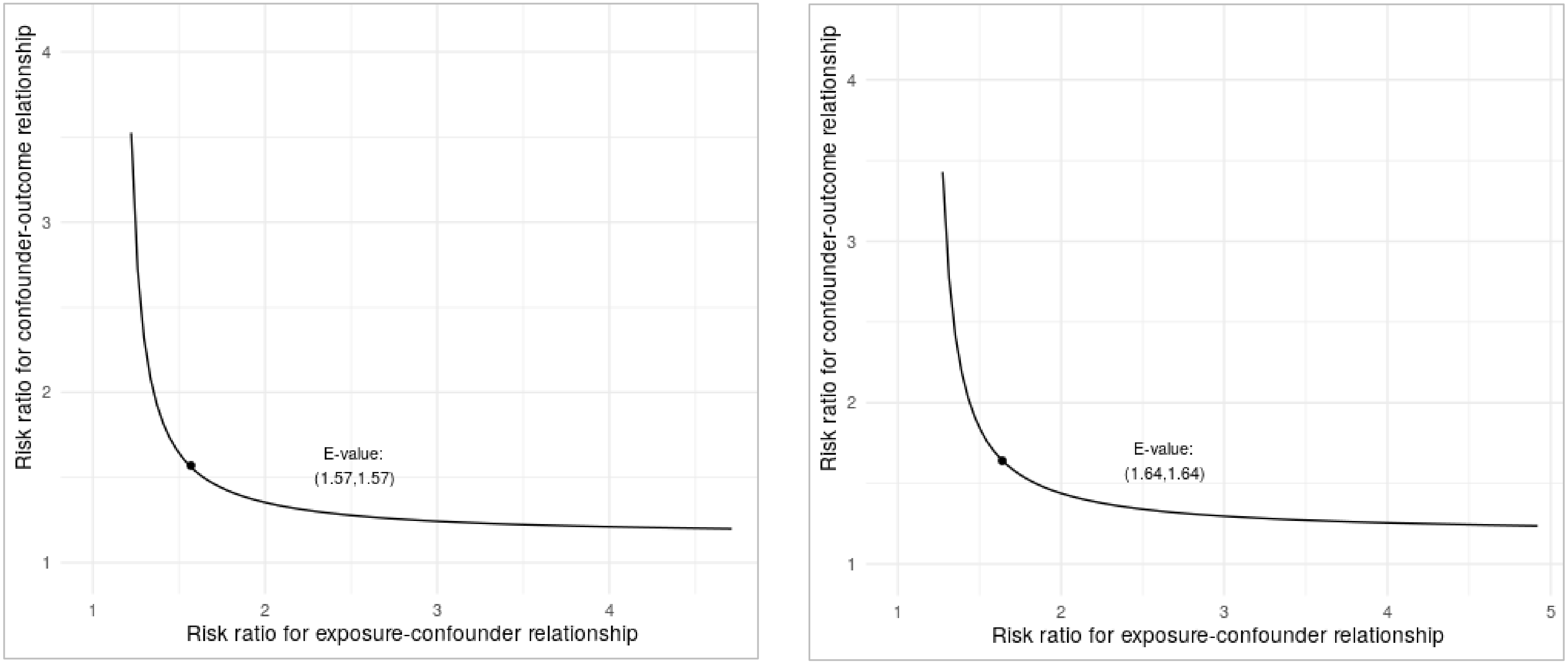

Under the assumptions of the E-value, to nullify the adjusted estimate for valproate in the adult or pediatric trial emulation, an unmeasured confounder would need to be associated with both the choice of mood stabilizer treatment and the onset of T2D by a magnitude of at least 1.6-fold, above and beyond the measured confounders.

Sensitivity analysis was conducted on the adjusted hazard ratio comparing valproate treatment to lamotrigine treatment, where the strongest magnitude of association was observed. The plots illustrate the magnitude of confounding needed to explain away the observed association. The x-axis reflects the range of risk ratios for the association between an unmeasured confounder and the choice of mood stabilizer treatment (exposure). The y-axis reflects the range of risk ratios for the association between an unmeasured confounder and type 2 diabetes (outcome). The E-value is the minimum strength of association (on the risk ratio scale) that an unmeasured confounder would need to have on both the choice of mood stabilizer treatment and the onset of type 2 diabetes (conditional on measured covariates) to fully explain away the observed association in the intention-to-treat analysis. While the E-value was originally proposed for the risk ratio, the same formula can be applied to hazard ratios with a rare outcome.[9] Results were generated using the online E-value calculator.[10]

© 2022 Sun JW et al. *JAMA Network Open.*

eTable 11. Sensitivity Analysis Evaluating the Potential Impact of Truncating Inverse Probability Weights

Treatment Group	Distribution of Weights			Adjusted Hazard Ratio (95% Confidence Interval)		
	Mean (SD)	Minimum	Maximum	Carbamazepine	Oxcarbazepine	Valproate
**Adult Trial**						
Intention-to-treat						
No truncation	1.02 (1.03)	0.12	91.84	1.02 (0.89-1.17)	1.06 (0.97-1.16)	1.14 (1.07-1.21)
0.5th and 99.5th percentile	1.00 (0.71)	0.17	5.86	1.07 (0.95-1.20)	1.07 (0.98-1.17)	1.15 (1.09-1.22)
1st and 99th percentile	0.99 (0.65)	0.18	4.37	1.07 (0.96-1.20)	1.07 (0.98-1.17)	1.16 (1.09-1.22)
5th and 95th percentile	0.94 (0.46)	0.41	2.19	1.13 (1.03-1.24)	1.09 (1.00-1.19)	1.19 (1.13-1.26)
Per-protocol						
No truncation	1.00 (1.88)	1.37×10^{-5}	1117.98	1.15 (0.80-1.66)	1.06 (0.89-1.27)	1.11 (0.97-1.26)
0.5th and 99.5th percentile	0.97 (0.74)	0.12	6.17	1.18 (0.92-1.49)	1.08 (0.91-1.28)	1.13 (1.00-1.27)
1st and 99th percentile	0.96 (0.67)	0.16	4.50	1.17 (0.93-1.46)	1.08 (0.91-1.29)	1.14 (1.01-1.28)
5th and 95th percentile	0.92 (0.47)	0.35	2.21	1.18 (0.98-1.43)	1.12 (0.95-1.32)	1.17 (1.05-1.31)
**Pediatric Trial**						
Intention-to-treat						
No truncation	1.00 (0.70)	0.16	24.98	1.01 (0.57-1.81)	1.09 (0.77-1.55)	1.19 (0.86-1.64)
0.5th and 99.5th percentile	1.00 (0.63)	0.37	4.78	1.02 (0.58-1.82)	1.10 (0.78-1.55)	1.20 (0.87-1.64)
1st and 99th percentile	0.99 (0.59)	0.39	3.79	1.04 (0.58-1.84)	1.11 (0.78-1.56)	1.18 (0.87-1.60)
5th and 95th percentile	0.95 (0.46)	0.46	2.17	1.07 (0.60-1.89)	1.09 (0.78-1.52)	1.11 (0.84-1.46)
Per-protocol						
No truncation	1.04 (7.78)	8.18×10^{-6}	1783.72	1.17 (0.45-3.01)	0.78 (0.40-1.53)	1.36 (0.73-2.52)
0.5th and 99.5th percentile	0.96 (0.80)	0.03	6.51	1.19 (0.46-3.06)	0.83 (0.43-1.60)	1.41 (0.76-2.67)
1st and 99th percentile	0.94 (0.73)	0.06	4.70	1.23 (0.48-3.14)	0.84 (0.44-1.61)	1.39 (0.75-2.56)
5th and 95th percentile	0.90 (0.53)	0.25	2.30	1.38 (0.55-3.46)	0.87 (0.46-1.63)	1.37 (0.78-2.41)

This analysis explored the bias-variance tradeoff in weight truncation. More truncation results in more biased point estimates, but smaller variance. In general, well behaved-weights have a mean of 1 and small range.[6] In the primary analysis, we truncated weights at the 1st and 99th percentile. Adjusted hazard ratios were weighted by the inverse probability of treatment and the inverse probability of censoring (adjusted for baseline covariates in the intention-to-treat analysis, adjusted for baseline and time-varying covariates in the per-protocol analysis).

© 2022 Sun JW et al. *JAMA Network Open.*

eTable 12. Sensitivity Analysis Evaluating the Potential Role of Different Grace Periods in the Per-Protocol Analysis

	No. of patients	No. of events	Total follow up in person-years	Mean follow up in person-years (SD)	Incidence rate per 1,000 person-years	Crude HR (95% CI)	Adjusted HR (95% CI)
**Adult Trial**							
100% Grace Period (Primary Analysis)							
Carbamazepine	26,641	220	9,193	0.35 (0.59)	23.93	1.55 (1.34-1.79)	1.17 (0.93-1.46)
Lamotrigine	132,739	1160	79,091	0.60 (0.79)	14.67	ref	ref
Oxcarbazepine	24,226	202	9,998	0.41 (0.60)	20.20	1.31 (1.13-1.53)	1.08 (0.91-1.29)
Valproate	90,600	803	36,988	0.41 (0.66)	21.71	1.44 (1.31-1.57)	1.14 (1.01-1.28)
200% Grace Period (Sensitivity Analysis)							
Carbamazepine	26,641	277	12,179	0.46 (0.70)	22.74	1.53 (1.35-1.75)	1.22 (1.00-1.49)
Lamotrigine	132,739	1406	99,578	0.75 (0.91)	14.12	ref	ref
Oxcarbazepine	24,226	248	13,024	0.54 (0.72)	19.04	1.29 (1.13-1.48)	1.09 (0.93-1.26)
Valproate	90,600	940	44,811	0.49 (0.74)	20.98	1.44 (1.32-1.56)	1.16 (1.05-1.29)
**Pediatric Trial**							
100% Grace Period (Primary Analysis)							
Carbamazepine	2,532	6	1,084	0.43 (0.63)	5.54	1.87 (0.81-4.34)	1.23 (0.48-3.14)
Lamotrigine	36,394	57	22,463	0.62 (0.79)	2.54	ref	ref
Oxcarbazepine	12,434	17	6,812	0.55 (0.75)	2.50	0.94 (0.54-1.61)	0.84 (0.44-1.61)
Valproate	22,645	30	7,990	0.35 (0.58)	3.75	1.23 (0.79-1.92)	1.39 (0.75-2.56)
200% Grace Period (Sensitivity Analysis)							
Carbamazepine	2,532	8	1,464	0.58 (0.80)	5.46	1.98 (0.95-4.12)	1.44 (0.66-3.15)
Lamotrigine	36,394	71	29,083	0.80 (0.92)	2.44	ref	ref
Oxcarbazepine	12,434	21	8,739	0.70 (0.87)	2.40	0.94 (0.57-1.52)	0.90 (0.51-1.59)
Valproate	22,645	33	9,811	0.43 (0.67)	3.36	1.14 (0.75-1.72)	1.28 (0.73-2.25)

One of the censorship events in the per-protocol analysis was treatment discontinuation. In the primary analysis, we allowed a gap between the end of supply and the next prescription filled that was equal to the days supplied of the current dispensing (e.g., for a 30-day dispensing, we allowed an additional 30 days between the end of supply and the next prescription filled; "100% grace period"). Treatment episodes were considered discontinued if the next prescription was not filled by the end of this allowable gap. In a sensitivity analysis, we allowed a gap that was equal to twice the days supplied of the current dispensing (e.g., for a 30-day dispensing, we allowed an additional 60 days between the end of supply and the next prescription filled; "200% grace period"). Inverse probability weights were truncated at the 1st and 99th percentiles. In the sensitivity analysis, the mean (SD)/range of the inverse probability weights were 0.96 (0.63)/0.18 to 4.27 in the adult trial emulation and 0.93 (0.66)/0.10 to 4.15 in the pediatric trial emulation.

© 2022 Sun JW et al. *JAMA Network Open.*

eReferences

1. Gagne JJ, Glynn RJ, Avorn J, Levin R, Schneeweiss S. A combined comorbidity score predicted mortality in elderly patients better than existing scores. *J Clin Epidemiol*. 2011;64(7):749-759. doi:10.1016/j.jclinepi.2010.10.004

2. Sun JW, Bourgeois FT, Haneuse S, et al. Development and Validation of a Pediatric Comorbidity Index. *Am J Epidemiol*. 2021;190(5):918-927. doi:10.1093/aje/kwaa244

3. Mansournia MA, Etminan M, Danaei G, Kaufman JS, Collins G. Handling time varying confounding in observational research. *BMJ*. 2017;359:j4587. doi:10.1136/bmj.j4587

4. Robins JM, Hernán MÁ, Brumback B. Marginal Structural Models and Causal Inference in Epidemiology: *Epidemiology*. 2000;11(5):550-560. doi:10.1097/00001648-200009000-00011

5. Hernán MÁ, Brumback B, Robins JM. Marginal Structural Models to Estimate the Causal Effect of Zidovudine on the Survival of HIV-Positive Men: *Epidemiology*. 2000;11(5):561-570. doi:10.1097/00001648-200009000-00012

6. Cole SR, Hernán MA. Constructing Inverse Probability Weights for Marginal Structural Models. *American Journal of Epidemiology*. 2008;168(6):656-664. doi:10.1093/aje/kwn164

7. Kalbfleisch JD, Prentice RL. *The Statistical Analysis of Failure Time Data*. Vol 360. John Wiley & Sons; 2011.

8. Murray EJ, Caniglia EC, Petito LC. Causal survival analysis: A guide to estimating intention-to-treat and per-protocol effects from randomized clinical trials with non-adherence. *Research Methods in Medicine & Health Sciences*. 2021;2(1):39-49. doi:10.1177/2632084320961043

9. VanderWeele TJ, Ding P. Sensitivity Analysis in Observational Research: Introducing the E-Value. *Ann Intern Med*. 2017;167(4):268-274. doi:10.7326/M16-2607

10. Mathur MB, Ding P, Riddell CA, VanderWeele TJ. Website and R Package for Computing E-Values. *Epidemiology*. 2018;29(5):e45-e47. doi:10.1097/EDE.0000000000000864

© 2022 Sun JW et al. *JAMA Network Open.*
